# Inflammasome‐induced extracellular vesicles harbour distinct RNA signatures and alter bystander macrophage responses

**DOI:** 10.1002/jev2.12127

**Published:** 2021-08-02

**Authors:** Christina F. Budden, Linden J. Gearing, Romina Kaiser, Lena Standke, Paul J. Hertzog, Eicke Latz

**Affiliations:** ^1^ Institute of Innate Immunity University Hospital University of Bonn Bonn Germany; ^2^ Department of Microbiology and Immunology The University of Melbourne at the Peter Doherty Institute for Infection and Immunity Melbourne Victoria Australia; ^3^ Centre for Innate Immunity and Infectious Diseases Hudson Institute of Medical Research Clayton Victoria Australia; ^4^ Department of Molecular and Translational Sciences Monash University Clayton Victoria Australia; ^5^ Department of Infectious Diseases and Immunology University of Massachusetts Medical School Worcester Massachusetts USA; ^6^ German Centre for Neurodegenerative Diseases (DZNE) Bonn Germany

**Keywords:** cell communication, extracellular vesicle, inflammasome, inflammation, interferon β, nucleotide‐binding oligomerization domain (NOD)‐containing protein with a pyrin domain (NLRP3), pyroptosis

## Abstract

Infectious organisms and damage of cells can activate inflammasomes, which mediate tissue inflammation and adaptive immunity. These mechanisms evolved to curb the spread of microbes and to induce repair of the damaged tissue. Chronic activation of inflammasomes, however, contributes to non‐resolving inflammatory responses that lead to immuno‐pathologies. Inflammasome‐activated cells undergo an inflammatory cell death associated with the release of potent pro‐inflammatory cytokines and poorly characterized extracellular vesicles (EVs). Since inflammasome‐induced EVs could signal inflammasome pathway activation in patients with chronic inflammation and modulate bystander cell activation, we performed a systems analysis of the ribonucleic acid (RNA) content and function of two EV classes. We show that EVs released from inflammasome‐activated macrophages carry a specific RNA signature and contain interferon β (IFNβ). EV‐associated IFNβ induces an interferon signature in bystander cells and results in dampening of NLRP3 inflammasome responses. EVs could, therefore, serve as biomarkers for inflammasome activation and act to prevent systemic hyper‐inflammatory states by restricting NLRP3 activation in bystander cells.

## 1 INTRODUCTION

Inflammation is a vital response of the immune system that evolved to limit microbial infections and is necessary to repair sterile tissue damage. The inflammatory processes typically subside, and normal tissue and organ function can resume after eliminating the infectious organisms and re‐establishing tissue homeostasis. However, if the immune response cannot stop the pathogen or if sterile triggers of inflammation persist, then chronic, non‐resolving inflammation develops and can result in inflammatory diseases.

Infectious organisms, cell stress or cell damage activate germline‐encoded innate immune signalling receptors, including toll‐like receptors (TLRs) and inflammasomes, through microbial signature molecules or the appearance of modified or mis‐localized host molecules. While TLR activation induces transcriptional reprogramming within cells and thereby the expression of pro‐inflammatory cytokines and type I interferons (IFNs) (Akira et al., 2006), inflammasomes control the post‐translational proteolytic activation of pro‐inflammatory cytokines (interleukin‐1β (IL‐1β) and IL‐18) and gasdermins, which together orchestrate a pro‐inflammatory form of cell death, termed pyroptosis (Broz, 2019). In the last two decades, basic research uncovered many of the activation mechanisms for the NLRP3 inflammasome, including the requirement for two signals to achieve pathway activation: (1) a priming signal, for example, delivered by lipopolysaccharide (LPS) and sensed by TLR4, which induces transcription of NLRP3 and pro‐IL‐1β, and (2) an activation signal, for example, delivered by crystals or pore‐forming toxins, which enables the formation of a proteolytically active inflammasome and hence culminates in the release of mature IL‐1β, IL‐18, and the induction of pyroptosis (Broz, 2019).

In addition to its immuno‐protective roles, activation of the NLRP3 inflammasome is increasingly recognized as a contributing factor in the pathogenesis of different inflammatory diseases (Duewell et al., 2010; Halle et al., 2008; Heneka et al., 2013; Masters et al., 2010). Additionally, gain‐of‐function mutations in NLRP3 cause a spectrum of cryopyrin‐associated periodic syndromes (CAPS) characterized by systemic inflammation (Hoffman et al., 2001). Studies in mice carrying these mutations demonstrate that NLRP3 overactivity leads to systemic inflammatory responses and pathologies in multiple organs (Brydges et al., 2009). Of note, IL‐1β and IL‐18 deficiency only partially rescue the otherwise rapidly lethal inflammatory phenotype (Brydges et al., 2013). At the same time, caspase 1 and gasdermin D are fully required for the systemic NLRP3‐mediated immuno‐pathologies to occur in this disease model (Xiao et al., 2018). These data show that caspase 1‐ and gasdermin D‐dependent factors are essential mediators of inflammasome‐dependent inflammation and could serve as valuable biomarkers of inflammasome activity.

The last decades of research have deciphered many of the proximal events of inflammasome activation. However, inflammasome downstream effects on the inflammatory response remain largely unclear, which hinders our understanding of immuno‐pathologies and represents a challenge for translating novel therapeutic approaches in broader and complex diseases.

One such consequence of inflammasome activation is the release of extracellular vesicles (EVs) (Cypryk et al., 2014; Lorey et al., 2017; MacKenzie et al., 2001; Mouasni et al., 2019; Öhman et al., 2014; Qu et al., 2007, 2009; Rühl et al., 2018; Sarkar et al., 2019; Valimaki et al., 2013; Zhang et al., 2017). As membranous compartments present in almost all body fluids, EVs represent important cell‐to‐cell communication mediators (Yáñez‐Mó et al., 2015). The EV content is protected from degradation and could, therefore, provide a useful biomarker indicating the upstream event that caused the EV release, such as inflammasome activation. EVs are commonly classified according to their cellular origin: apoptotic bodies released as blebs of apoptotic cells, ectosomes formed by outward budding of the plasma membrane, and exosomes derived from multi‐vesicular bodies (Mittelbrunn & Sánchez‐Madrid, 2012). EVs can carry a set of functional proteins and ribonucleic acids (RNAs), which differs from the healthy condition in multiple pathologies (Yuana et al., 2013). Additionally, the transfer of functional RNAs from an EV donor cell enables transcriptional changes in recipient cells (Valadi et al., 2007).

Thus, we here studied inflammasome‐induced EVs for two purposes: (1) to characterize the EV content upon inflammasome activation to elucidate novel EV‐associated biomarkers for pathway engagement, and (2) to characterize the functional effects of inflammasome‐induced EVs on bystander cells to provide novel mechanistic insight into inflammatory responses downstream of inflammasome activation.

Therefore, we characterized the transcriptional cargo of EVs released by human macrophages upon stimulation with multiple inflammatory triggers, including inflammasome and TLR stimuli. We identify a conserved RNA content induced by different NLRP3 activators, here defined as an EV‐associated NLRP3 signature, which differed from the RNA content of TLR‐induced EVs and, therefore, may have clinical relevance for biomarker development. Additionally, we discovered that NLRP3‐induced EVs contained IFNβ protein, which induced interferon‐stimulated genes (ISGs) in recipient macrophages and could limit inflammasome responses in un‐primed EV recipient macrophages.

## 2 MATERIALS AND METHODS

### 2.1 Cell lines

Wildtype (WT) THP‐1 monocytes were obtained from ATCC. Veit Hornung (University of Munich) kindly provided NLRP3 knockout (KO) THP‐1 monocytes, and we reconstituted these cells with human NLRP3. Seth Masters (University of Melbourne) provided THP‐1 monocytes expressing doxycycline‐inducible gasdermin D guide RNAs. We immortalized murine macrophages (iMos) as reported previously (De Nardo et al., 2018).

### 2.2 Differentiation and stimulation of THP‐1 monocytes

We differentiated THP‐1 monocytes with 100 nM PMA (Sigma) for 12–16 h, after which the cells were washed once with PBS and rested for 24 h before stimulation. For inflammasome activation, THP‐1 macrophages were primed with 200 ng/ml LPS (LPS‐EB Ultrapure; Invitrogen) in RPMI 10% FCS 1% PenStrep (ThermoFisher) for 2 h. After that, the cells were washed three times with PBS to remove the EVs present in FCS and LPS. Depending on the experiment, cells were pre‐treated with 5 μM CRID3 (Pfizer), 30 or 50 μM VX765 (Selleckchem), or DMSO (AppliChem) in serum‐free RPMI for 30 min. Inflammasome activators were added as follows: 10 μM nigericin (90 min; Invitrogen), 20 μg/ml R837 (120 min; Invivogen), 2.5 μM IFM‐680 (90 min, hereafter referred to as IFM; IFM Therapeutics), 500 μg/ml MSU crystals (120 min), 0.15 ng/ml PrgI (kindly provided by Matthias Geyer, University of Bonn) with 1 μg/ml protective antigen (PA; 90 min; List Biological Laboratories), 20 μg/ml R848 (120 min; Invivogen). For TLR stimulation, we washed and pre‐treated the cells as described for inflammasome activation. TLR stimuli were added as follows: 20 μg/ml R848 (24 h; Invivogen), 1.25 ng/ml Pam3CSK4 (P3CSK4, 24 h; Invivogen). Depending on the experiment, we used a total of 1 ⋅ 10^5^–30 ⋅ 10^6^ cells per stimulus.

### 2.3 Isolation, differentiation, and stimulation of primary human cells

We isolated primary human cells from buffy coats using Ficoll (GE Healthcare Life Sciences) gradient centrifugation to obtain peripheral blood mononuclear cells (PBMCs). We selected monocytes with CD14 microbeads (Miltenyi) and used rhGM‐CSF (500 U/ml; Immuno‐tools) to differentiate the monocytes to macrophages for 3 days. Primary human monocyte‐derived macrophages (hMDMs) were primed with 200 ng/ml LPS for 2 h, washed thrice with PBS, and subsequently stimulated with 10 μM nigericin for 60 min. Depending on the experiment, hMDMs were incubated with THP‐1 EVs before or after priming.

### 2.4 Isolation and differentiation of mouse bone marrow‐derived macrophages (BMDMs)

BMDMs were obtained after culturing bone marrow cells from WT, STING KO, cGAS KO, or UNC93B KO C57BL/6 mice in DMEM containing 10% FCS and 20% L929 cell‐conditioned medium.

### 2.5 Isolation of EVs from tissue culture supernatant

The EV isolation protocol was developed based on previous reports (Théry et al., 2006). Tissue culture supernatant was centrifuged at 340 *g* for 10 min to remove floating cells, 2000 g for 20 min to pellet 2K EVs, and 10,000 g for 40 min to pellet 10K EVs. We washed the 2K and 10K pellets in PBS before analysis. The supernatant was filtered using a 0.22 μm filter (Millipore) and then concentrated to a total volume of 0.5 ml using a 10K NMWL filter (Amicon Ultra, Sigma‐Aldrich). The sample was then transferred onto a size exclusion chromatography (SEC) column (qEV original, Izon) and we collected 0.5 ml fractions. The SEC EVs referred to within this study comprise the pooled fractions 7, 8, and 9. Depending on the experiment, SEC EVs were concentrated by centrifugation at 100,000 *g* for 90 min.

### 2.6 Isolation of EVs from blood plasma

We took blood from healthy donors using citrated tubes (S‐Monovette 9NC, Sarstedt) and a 21‐gauge needle. Blood was centrifuged at 1000 *g* for 15 min to obtain plasma. Plasma was centrifuged at 340 *g* for 10 min to remove all remaining cells, twice at 2500 g for 15 min to clear it of platelets, and at 10,000 g for 45 min to pellet 10K EVs. The 10K EVs were washed in PBS before analysis. The supernatant was concentrated to a total volume of 0.5 ml using a 10K NMWL filter (Amicon Ultra, Sigma‐Aldrich). The sample was then transferred onto a size exclusion chromatography column (qEV original, Izon) and fractions were collected as described above. SEC EVs were filtered through a 0.22 μm filter (Millipore) and centrifuged at 100,000 g for 90 min.

### 2.7 EV size and concentration measurements

EV size and concentration were measured by nanoparticle tracking analysis (NTA) using the NanoSight NS300 (Malvern). EVs secreted by 10 ⋅ 10^6^ THP‐1 macrophages were measured. 2K and 10K EVs were diluted in a total volume of 1 ml each. SEC EVs had to be diluted depending on the stimulus. Samples were applied to the NanoSight using an automated syringe pump. The syringe pump speed was set to 20 and the temperature was kept constant at 25°C. Per sample, three videos of 1 min each were recorded. Depending on the EV class analysed, the camera level was set to 9 (2K and 10K) or 14 (SEC). For analysis, particle tracks of each particle above the threshold, which was set to 20 (2K), 10 (10K), or 5 (SEC), were determined and correlated to a specific particle size.

### 2.8 Transmission electron microscopy of EVs

Carbon‐coated formvar copper grids were rendered hydrophilic by glow discharging for 2 min. 5 μl of isolated EVs resuspended in PBS were transferred onto the grid. Grids were washed thrice with water and subsequently stained with 2% aqueous uranyl acetate for 1 min and again washed with water. Grids were carefully blow‐dried and analysed on a 200 kV transmission electron microscope (LEO 992 A EFTEM Zeiss) with Troendle SCCD Detector (2k × 2k).

### 2.9 Isolation and quantification of vesicular and cellular RNA

For RNA subjected to the Clariom D microarray, the miRCURY RNA Isolation Kit – Cell and Plant (Exiqon) was used according to the manufacturer's instructions. Subsequently, DNase digest was performed using the Turbo DNA‐free kit (Invitrogen). Since the Exiqon kit was later discontinued, the RNeasy Plus Micro Kit (Qiagen) was used according to the manufacturer's instructions for all qRT‐PCRs and transfer experiments.

RNA concentration measurements were performed either with the Qubit RNA HS assay kit (ThermoFisher) or with the 2200 Tape Station System using the High Sensitivity RNA Screen Tape Sample Buffer (Agilent).

### 2.10 cDNA production and qRT‐PCR

RNA subjected to cDNA transcription was normalized to 300 ng. The volume was adjusted to 12.9 μl using water, 1 μl oligo dT was added, and the sample was heated to 65°C for 5 min. Samples were incubated on ice for 1 min, and 4 μl 5× reaction buffer, 1 μl 10 mM dNTPs, 1 μl 0.1 M DTT, and 0.1 μl SuperScript III (ThermoFisher) were added. Reverse transcription was performed at 50°C for 50 min and finalized by heating to 85°C for 5 min. cDNA was diluted 1:5 for qRT‐PCR. Per sample, 5 μl Maxima, SYBR Green/ROX qPCR Master Mix (ThermoFischer), 2 μl 2 μM primer mix of forward and reverse primer and 1 μl water were added to 2 μl diluted cDNA. Each sample was run in duplicates on a QuantStudio 6.2 (ThermoFischer).

The following primers were used (fw = forward, rev = reverse):

hMX2 fw: GAACGTGCAGCGAGCTTGTC; hMX2 rev: GTAGGGCCAAGGCTTGTGGG;

hISG15 fw: GCTGAGAGGCAGCGAACTCA; hISG15 rev: CGCCAGCATCTTCACCGTCA;

hOAS3 fw: GCTGGTCACCCAGTACCGC; hOAS3 rev: GGATGATAGGCCTGGGCTTCTG;

hIFI35 fw: CCAGGTGATGATGTCCAGCCAG; hIFI35 rev: CCACATCGCCACCTCCGTTC;

hLY6E fw: TTGGTTTGTGACCTCCAGGCAG; hLY6E rev: AGCAGGAGAAGCACATCAGCG;

hIFITM3 fw: TGCTGATCTTCCAGGCCTATGGA; hIFITM3 rev: GGCAGGGCGAGGAATGGAAG;

hIFI6 fw: GCTCCGGGCTGAAGATTGCT; hIFI6 rev: TTACCTGCCTCCACCCCACT;

hBST2 fw: TCTGCAGAGGTGGAGCGACT; hBST2 rev: GAGGCCCAGCAGCACAATCA;

hTNF fw: CCCAGGCAGTCAGATCATCTTC; hTNF rev: TCTCTCAGCTCCACGCCATT;

hIL6 fw: GGTACATCCTCGACGGCATCT; hIL6 rev: GTGCCTCTTTGCTGCTTTCAC;

mMx2 fw: ACCGTGGACGAGAATTGCCA; mMx2 rev: ACAATTTCAGTGACCGTGTGCAG;

mIsg15 fw: CAATGGCCTGGGACCTAAAG; mIsg15 rev: TAAGACCGTCCTGGAGCACT;

mOas3 fw: GCTTGCCAAGGAGGCTACCG; mOas3 rev: ACTTCACACAGCGGCCTTTACC;

mIfi35 fw: TGCTGATGAAGAAGTGGCCCAG; mIfi35 rev: CTGAATGAGGGGCTTGCTGGA;

mIfitm3 fw: GCCTACGCCTCCACTGCTAAG; mIfitm3 rev: GGACCGGAAGTCGGAATCCTCTA;

mBst2 fw: CTGTAGAGACGGGTTGCGAGC; mBst2 rev: CTCCTGAAGGGTCACCACGG

### 2.11 Transfer of EVs

Extracellular vesicles were transferred to recipient cells at a 40:1 EV donor to EV recipient cell ratio. EVs were co‐incubated with recipient cells for 15–20 h in RPMI with 10% FCS, 1% PenStrep, which had previously been centrifuged at 100,000 *g* for 18 h to remove bovine EVs. EV concentration and time of co‐incubation were determined by confocal microscopy with fluorescently labelled EVs (data not shown). Before RNA isolation, supernatants containing EVs were removed, and cells were washed three times with PBS to remove attached EVs.

### 2.12 Immunoblot

#### 2.12.1 LI‐COR instrument

Cells or EVs were lysed with RIPA buffer (20 mM Tris‐HCl, 150 mM NaCl, 1 mM EDTA, 1% Triton X‐100, 10% glycerol, 0.1% SDS, 1 mM sodium deoxycholate, 1x cOmplete EDTA‐free protease inhibitor (Roche Life Science), 0.2 mM PMSF). Protein concentration was determined using the Bichinoic acid assay kit (ThermoScientific). Samples were reduced and denatured by adding NuPAGE LDS Sample Buffer (4x) and NuPAGE Sample Reducing Agent (10x) and heating at 85°C for 10 min. 30 μg total cell lysate or the whole preparation of EVs were run on a 4–12% Bis‐Tris gel (Novex; Invitrogen) with MES buffer (Novex; Invitrogen) and transferred to Immobilon‐FL PVDF membranes (Millipore). Non‐specific binding was blocked with 3% BSA in Tris‐buffered saline for 1 h, followed by overnight incubation with specific primary antibodies in 3% BSA in Tris‐buffered saline with 0.1% Tween‐20.

#### 2.12.2 WES instrument

Cells or EVs were lysed with RIPA buffer, and protein concentration was determined as described above. Samples were reduced by adding DTT (anti‐CD9 and anti‐CD81 required non‐reducing conditions). WES (ProteinSimple) was performed with 2 μg protein per capillary according to the manufacturer's instructions.

The following primary antibodies were used:

anti‐β‐actin (926‐42212; LI‐COR), anti‐AGO2 (ab32381; Abcam), anti‐calnexin (MAB3126; Merck), anti‐gasdermin D (126‐138; Sigma), anti‐histone H3 (D1H2, 4499T; Cell Signalling), anti‐TSG101 (4A10; Thermo Scientific), anti‐CD9 (Ts9; Invitrogen), anti‐CD81 (M38; Invitrogen), anti‐HSP70 (N27F3‐4; CST)

### 2.13 Cytokine quantification by homogenous time‐resolved fluorescence (HTRF)

The assays (human IL‐1β HTRF, human TNFα HTRF, and human IFNβ HTRF; cisbio) were performed according to the manufacturer's instructions to quantify cytokines. For IFNβ quantification in supernatants, supernatants were concentrated using a 10K NMWL filter (Amicon Ultra, Sigma‐Aldrich) thereby accounting for the concentration factor of EVs and making IFNβ concentrations in EVs and supernatants comparable. For quantification in EVs, EVs were either used intact or lysed in 0.1% Triton X‐100.

### 2.14 Cell viability assays

#### 2.14.1 Lactate dehydrogenase (LDH) assay

The Pierce LDH Cytotoxicity Assay (Life Technologies) was performed according to the manufacturer's instructions. To obtain the percentage of dead cells, values measured were normalized to the maximal LDH release controls, which equals 100% cell death.

#### 2.14.2 CellTiter‐Blue (CTB) assay

Once the cell supernatants for cytokine detection and EV isolation had been harvested, 50 μl medium containing 10% CTB reagent (Promega) was added per 96‐well. After incubation at 37°C for 30 min to 1 h, fluorescence was measured according to the manufacturer's instructions.

### 2.15 Endotoxin quantification assay

The Pierce chromogenic endotoxin Quant kit (ThermoFisher) was used according to the manufacturer's instructions.

### 2.16 Clariom D microarray analysis: THP‐1 EVs and blood plasma EVs

Five replicates of each sample were generated, that is, 110 samples were subjected to a Clariom D Pico microarray performed in the ThermoFisher laboratories in San Diego. Microarrays were processed in four batches. We removed 17 samples from the analysis due to failed mid‐assay quality controls or scan issues. The input RNA amount per sample was 1 ng for 87 samples and < 1 ng for six samples. Due to the lack of samples from EVs released after 90 min LPS priming (Figure S3a), all analyses involving priming‐only EVs used the 120 min time point.

Raw intensity values, CHP files containing signal space transformation‐robust multi‐array average (SST‐RMA) normalized data, as well as transcript annotation, were provided by ThermoFisher. Additional probe annotation was obtained using the Bioconductor clariomdhumantranscriptcluster.db package v8.7. Normalized expression values were extracted from the CHP files and then processed using the limma package v3.38.2 (Ritchie et al., 2015). Only main category probes were retained, and, as recommended by ThermoFisher, probes with more than one match to the transcriptome were removed from the analysis. A batch effect, corresponding to the microarray processing batch, was incorporated into the design matrix. Quality weighting was applied to each sample using the array Weights function (Ritchie et al., 2006). Moderated *t*‐statistics were calculated using the treat function (Mccarthy & Smyth, 2009) with the fold change (FC) threshold set to 1.1 (THP‐1 EVs) or 1.2 (blood plasma EVs). For each comparison, significantly differentially abundant transcripts were identified with Benjamini‐Hochberg (BH)‐adjusted *P*‐value < 0.05.

Gene set testing was performed using either the camera function of the limma package v3.38.2 (Wu & Smyth, 2012) or the egsea.ora function of the EGSEA package v1.10 (Alhamdoosh et al., 2017) with the hallmark and gene ontology (GO) gene set collections from the Molecular Signatures Database v5.2 (Liberzon et al., 2015; Subramanian et al., 2005).

For the definition of NLRP3 and inflammasome signatures, transcripts were identified that were consistently significantly up‐ and down‐regulated for the comparisons of interest. To assess transcript types, gene biotypes were extracted from Ensembl BioMart (Kinsella et al., 2011) using the biomaRt package v2.38. If there was no biotype annotation for a transcript, the annotation was taken from the locus type information provided by ThermoFisher if available, else the transcript type was annotated as ‘other’.

### 2.17 3′ mRNA‐sequencing analysis

RNA integrity was assessed using the Agilent 2100 Bioanalyzer. The minimum cellular RNA integrity number (RIN) across all samples was 9.7. All 100 samples were transferred to the Next Generation Sequencing Core Facility of the Medical Faculty of the University of Bonn for library preparation using the QuantSeq 3′ mRNA Library Prep Kit FWD for Illumina, according to the manufacturer's instructions. Samples were processed in three batches. RNA libraries were prepared with 50 ng of total cellular RNA or 30 ng of total vesicular RNA.

We performed 3′ sequencing on the Illumina Hiseq2500 with an average of 20 · 10^6^ reads per sample. Raw reads were trimmed and Illumina adaptor sequences removed using Trimmomatic v0.36 (Bolger et al., 2014). Raw reads were aligned to the human reference genome GRCh38 using STAR software v2.5.3a_modified (Dobin et al., 2012). The biomaRt package v2.38.0 (Kinsella et al., 2011) was used to obtain gene names and Entrez IDs. Counts were processed using the edgeR package v3.24.0 (Robinson et al., 2010). Genes were analysed if they were detected in at least five samples, with at least 1.76 counts per million (cpm). This threshold was calculated by determining the cpm value of 10 raw counts using the median library size. Normalization factors were determined using the TMM method (Robinson & Oshlack, 2010). A batch effect, corresponding to the sequencing processing batch, was incorporated into the design matrix. The edgeR estimateDisp function stating the design matrix was used to estimate the common and tagwise dispersion, and to convert counts to weighted log_2_ counts per million expression values. Robust was set to TRUE. The glmQLFit function with robust set to TRUE and the glmQLFTest function were used to determine which genes were differentially expressed in each comparison. Differentially expressed transcripts were identified with false discovery rate (FDR)‐adjusted *P*‐value < 0.05.

Gene set testing was performed as for the Clariom D Microarrays. Genes of interest were compared to the Interferome database v2.0 (Rusinova et al., 2013), and their abundance across all Interferome data sets was determined.

For transcription factor binding site (TFBS) predictions, the promoter regions of significantly changed genes in the comparison of interest were compared to the promoter regions of genes with log_2_(FC) < 0.2 in the same comparison. The region to be analysed for TFBSs was set to 400 bases upstream and 100 bases downstream of the transcription start site (TSS, identified using Ensembl v95). Predictions were made using three different tools. CiiiDER (Gearing et al., 2019) analyses were conducted using the human GRCh38 genome with the 2018 JASPAR core non‐redundant vertebrate matrices (Khan et al., 2018) and with a deficit of 0.15. AME (Mcleay & Bailey, 2010) analyses (MEME suite v5.0.5) were performed using the 2018 JASPAR core non‐redundant vertebrate matrices. The sequence scoring method was set to average odds score, and the motif enrichment test was set to Fisher's exact test. The E‐value threshold for reporting enriched motifs was adjusted to 1000. t‐distributed stochastic neighbour embedding (t‐SNE) was performed with the Rtsne package v0.15 (perplexity 50) using transcription factor (TF) matrix clustering distances downloaded from the JASPAR website. HOMER v4.9.1 (Heinz et al., 2010) analyses were performed using the HOMER vertebrate motif collection and the findMotifs.pl function to look for enrichment of known motifs but not for *de novo* motif enrichment.

### 2.18 Data availability

Gene expression data was uploaded to GEO with SuperSeries accession number GSE180709. Raw and processed expression data from the Clariom D microarrays of THP‐1 EVs were deposited with SubSeries accession number GSE180708. For the 3′ sequencing, the raw FASTQ files and gene counts generated by STAR were deposited with SubSeries accession number GSE180707.

### 2.19 Statistical analysis

Pooled data from a minimum of three independent experiments or three individuals are typically depicted as mean + standard error of the mean (SEM). Otherwise, data are shown as mean + standard deviation (SD). Statistical analysis was performed using GraphPad Prism and R. We assessed statistical significance for transcriptomic data as described above and for all other data by ANOVA or paired *t*‐test using the original FDR method of Benjamini and Hochberg or the Dunnett test for *P*‐value adjustment.

### 2.20 Ethics

Human monocytes were extracted from blood concentrates provided by the blood donation service of the University Hospital Bonn (ERC Ethikantrag Lfd. Nr. 184/16 ‘Activation and regulation of Inflammasomes (InflammAct)’ and Ethikantrag Lfd. Nr. 392/20 ‘Antrag zur Verwendung von Buffy Coats und Vollblut am Institut für Angeborene Immunität’). Our study complied with all relevant ethical regulations for animal testing and research.

## 3 RESULTS

### 3.1 EV release upon NLRP3 activation temporally correlates with IL‐1β release

**FIGURE 1:**
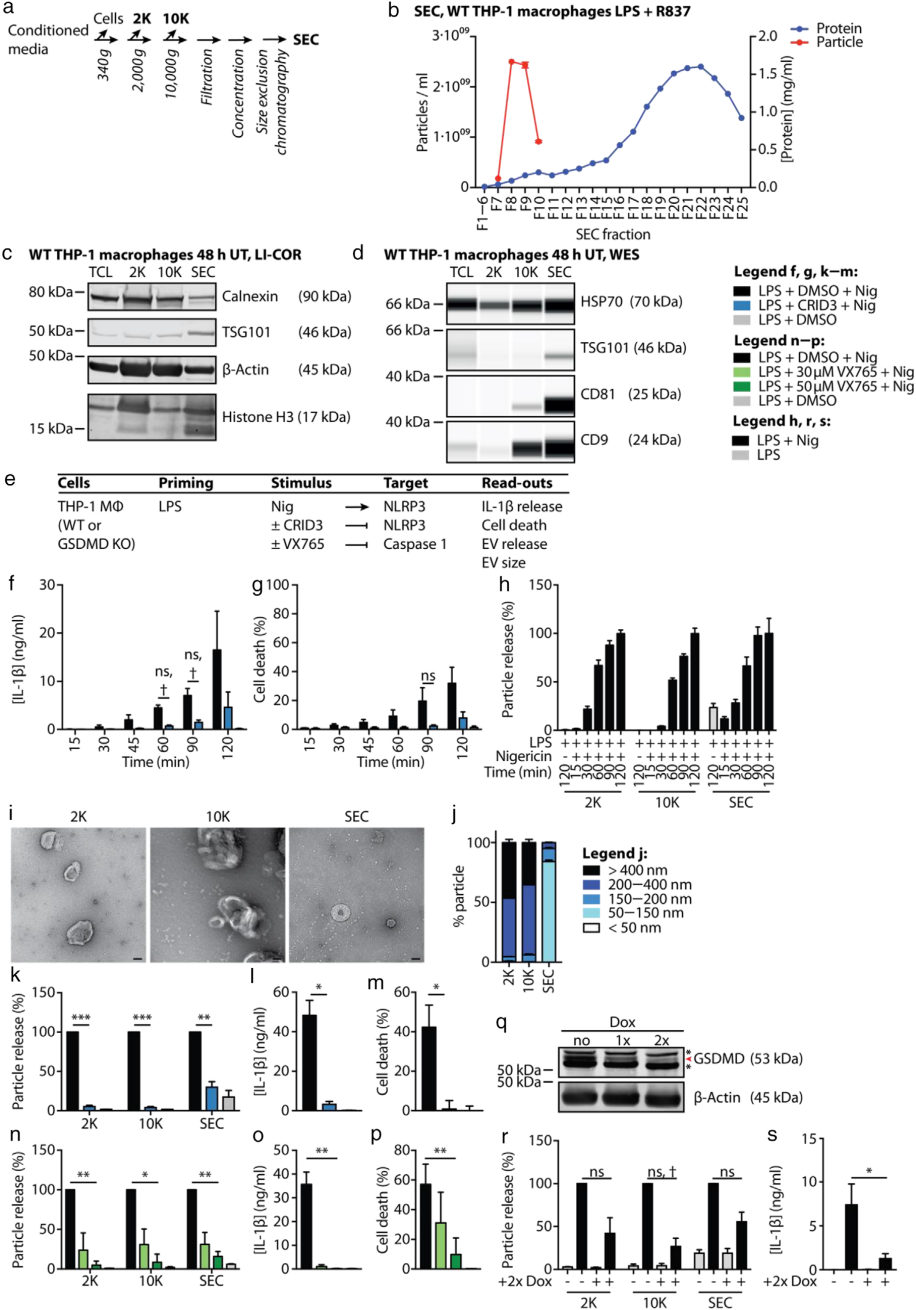
EV release upon NLRP3 activation with LPS + nigericin temporally correlates with IL‐1β release and is an NLRP3‐, caspase 1‐, and gasdermin D‐dependent event. (a), Schematic diagram of the EV isolation protocol used in subsequent experiments to obtain three distinct EV classes: 2K, 10K, and SEC EVs. (b), PMA‐differentiated WT THP‐1 macrophages were stimulated with 200 ng/ml LPS for 120 min and subsequently treated with 20 μg/ml R837 for 120 min to induce EV release. NTA and protein concentration measurements were performed on fractions eluting from a size exclusion chromatography (SEC) column. Representative experiment of *n* = 2. (c, d), PMA‐differentiated WT THP‐1 macrophages were left unstimulated for 48 h in serum‐free medium. Immunoblot of 30 μg protein (c, LI‐COR, representative experiment of *n* = 2) or 2 μg protein (d, WES, representative experiment of *n* = 3) per EV class. TCL: total cell lysate. (e), Table summarizing cells, their stimulation and the read‐outs performed in experiments depicted in f−s. MΦ: macrophage. (f−p), 10 · 10^6^ PMA‐differentiated WT THP‐1 macrophages were primed with 200 ng/ml LPS for 120 min, depending on the experiment pre‐incubated with 5 μM CRID3, 30 or 50 μM VX765, or the vehicle DMSO, and subsequently treated with 10 μM nigericin for 90 min, unless otherwise specified. IL‐1β release into the tissue culture supernatant was determined by HTRF (f, l, o), cell death levels were determined measuring LDH release (g, m, p). Particle counts and size were determined using NTA. Relative particle counts were either normalized to the particle count upon LPS + nigericin treatment (h) or normalized to the particle count upon LPS + DMSO + nigericin treatment (k, n). For particle size distributions, particle counts were normalized to the total number of particles measured in each EV class (j). To visualize EVs, they were transferred to a carbon‐coated copper grid, stained with 2% aqueous uranyl acetate and subjected to transmission electron microscopy. Scale bar = 100 nm (i). q, doxycycline (Dox)‐inducible gasdermin D (GSDMD) KO THP‐1 monocytes were treated with 1 μg/ml doxycycline for 72 h, either once (1x Dox) or twice with a 24 h rest in fresh RPMI in between (2x Dox). Subsequently, cells were subjected to immunoblot; asterisks indicate non‐specific bands. r, s, 10 · 10^6^ PMA‐differentiated doxycycline‐inducible gasdermin D KO THP‐1 macrophages per condition were primed with 200 ng/ml LPS for 120 min and subsequently stimulated with 10 μM nigericin for 90 min. Particle counts were determined using NTA. Relative particle counts were normalized to the particle count upon LPS + nigericin treatment in no Dox cells (first black bar) in each EV class (r). IL‐1β release into the tissue culture supernatant was determined by HTRF (s). (f, g, j−p, r, s), Pooled data from *n* = 3, each in technical triplicates, mean + SEM. h, Representative experiment from *n* = 2, each in technical triplicates, mean + SD. ns: not significant, †: unadjusted *P*‐value < 0.05, *: *P‐*value < 0.05, **: *P‐*value < 0.01, ***: *P‐*value < 0.001, ****: *P‐*value < 0.0001

We isolated EVs from conditioned media of THP‐1 macrophages based on their size, enriching large (2K), intermediately sized (10K), and small (size exclusion chromatography (SEC)) EVs (Figure 1a,b). SEC EVs were enriched in the exosome markers TSG101, CD9, and CD81 and depleted of the ER marker calnexin and all EV classes were positive for β‐actin, histone H3, and HSP70 (Figure 1c,d).

Since we were particularly interested in EVs released upon activation of the NLRP3 inflammasome, we primed THP‐1 macrophages with LPS followed by stimulation with the well‐characterized NLRP3 activators nigericin or R837 (Groß et al., 2016; Mariathasan et al., 2006) (Figures 1e and S1a). Expectedly, this stimulation induced an NLRP3‐dependent release of IL‐1β (Figures 1f and S1b) and pyroptosis (Figures 1g and S1c) over time. We observed that NLRP3 inflammasome activation additionally led to an increased release of 2K, 10K, and SEC EVs over time (Figures 1h and S1d).

To characterize inflammatory EVs more broadly, we tested multiple stimuli for their capacity to induce EV release (Figure S2a). For these studies, we used four NLRP3 activators (LPS + nigericin, LPS + R837, LPS + IFM, and LPS + MSU), one NLRC4 activator (LPS + PrgI) and two TLR ligands (R848 and P3CSK4). Since R837 has been shown to activate TLR7 (Lee et al., 2003) but also NLRP3 (Groß et al., 2016), we also included the structurally related but monospecific compounds R848 (specific for TLR7/8) and IFM (specific for NLRP3) in our studies. Additionally, we analysed EVs released from untreated (UT) or solely LPS‐primed THP‐1 macrophages. For simplicity, EVs released upon stimulation of EV donor cells are collectively referred to as EVs_stimulus_ or individually termed 10K_stimulus_ or SEC_stimulus_.

The 2K, 10K, and SEC EVs released upon NLRP3 stimulation (EVs_NLRP3_) showed a cup‐shaped morphology when visualized by electron microscopy (Figures 1i and S1e). This is a typical artefact of lipid bilayer‐surrounded material, such as EVs, occurring due to dehydration upon sample fixation (Brouwers et al., 2013). Nanoparticle tracking analyses revealed that the size distributions of 2K, 10K, and SEC EVs were comparable across all stimuli of interest and corresponded to the published size profiles of apoptotic bodies, ectosomes, or exosomes, respectively (Figures 1j, S1f and S2b—g,af,ag). While we did not formally investigate the cellular origin of EVs within this study, these data suggest that inflammasome activation results in the liberation of different EV classes.

### 3.2 EV release upon NLRP3 activation is an NLRP3‐, caspase 1‐, and gasdermin D‐dependent event

Next, we stimulated EV donor cells in the presence of the NLRP3 inhibitor CRID3 or the caspase 1 inhibitor VX765 to characterize whether the EV release was NLRP3‐ or caspase 1‐dependent. Gasdermin D‐dependency of EV release was further evaluated in doxycycline‐inducible gasdermin D KO THP‐1 macrophages (Figure 1q). NLRP3‐ and caspase 1‐dependent IL‑1β release and pyroptosis upon stimulation with nigericin (Figure 1l,m,o,p), R837 (Figure S1h,i,k,l), and IFM (Figure S2i,j) confirmed a successful NLRP3 stimulation in the cells. Additionally, the release of EVs_LPS+Nig_, EVs_LPS+R837_, and EVs_LPS+IFM_ was NLRP3‐ and caspase 1‐dependent (Figures 1k,n, S1g,i and S2h) and also partially dependent on gasdermin D (Figures 1r,s and S1m,n). In line with crystals inducing inflammasome‐independent cell death (Rashidi et al., 2020), MSU‐induced IL‐1β release and pyroptosis were only partially NLRP3‐ and caspase 1‐dependent, and they were not gasdermin D‐dependent (Figure S2l,m,aa). Similarly, the release of EVs_LPS+MSU_ was NLRP3‐, caspase 1‐, and gasdermin D‐independent (Figure S2k,z). NLRC4 activation by the type III secretion system needle complex protein PrgI induced caspase 1‐dependent and partially gasdermin D‐dependent, but NLRP3‐independent EV release (Figure S2n–p,ab,ac).

In contrast to the inflammasome activators mentioned above, the release of EVs_TLR_ was mostly independent of NLRP3, caspase 1, and gasdermin D (Figure S2q–y,ad,ae).

In summary, the majority of NLRP3 activators, except for MSU, induced EV secretion downstream of NLRP3, caspase 1, and gasdermin D. However, stimulation with TLR ligands led to NLRP3‐ and caspase 1‐independent EV release.

### 3.3 Transcripts contained within inflammasome and TLR EVs largely differ

**FIGURE 2:**
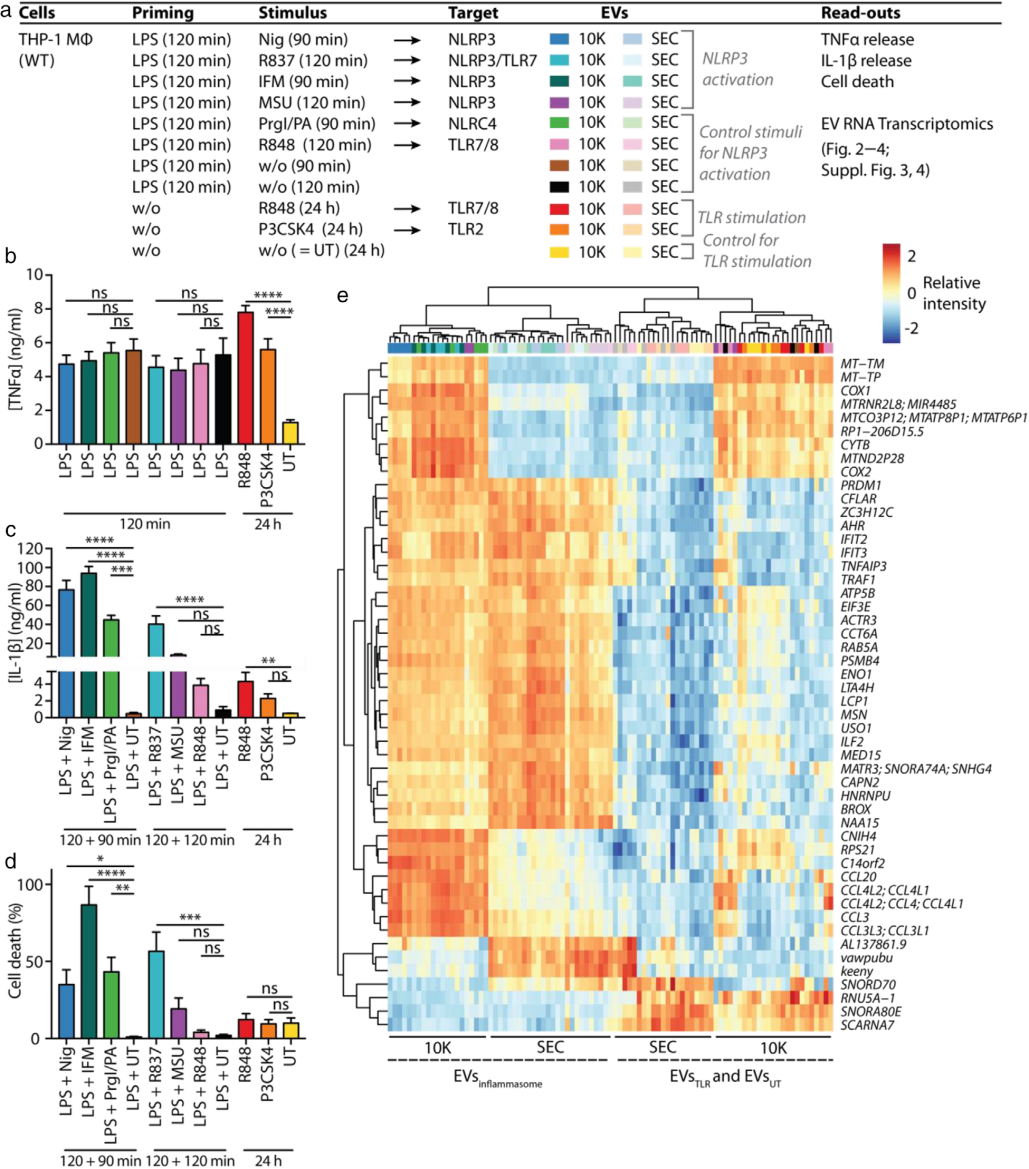
Characterization of EV content according to EV class and EV‐inducing stimulus. (a), Table summarizing cells, their stimulation and the read‐outs performed in experiments depicted in (b—e), Figures 3, 4, S3 and S4. MΦ: macrophage, w/o: without. Per condition, 3 · 10^7^ PMA‐differentiated WT THP‐1 macrophages were primed with LPS and subsequently treated with an inflammasome activator, primed only, TLR‐stimulated, or left untreated (UT) as indicated. 10K and SEC EVs from all conditions were purified from the tissue culture supernatant. Vesicular RNA was extracted and Clariom D Pico microarrays were performed. (b—d), Tissue culture supernatants were taken after priming or TLR stimulation (b) and after full stimulation to determine cytokine concentrations by HTRF (c) and cell death levels by LDH release (d). Pooled data of *n* = 5, each in technical triplicates, mean + SEM. (e), Heat map depicting relative expression values scaled to each row (to have mean zero and SD one) of the top 50 most variable coding transcripts across all samples. Multiple transcript labels per row indicate transcripts which cannot be distinguished from each other with the probe set used. Lines indicating samples derived from inflammasome EVs and TLR/UT EVs are dashed to indicate that sample clustering was not perfect, meaning that the majority of inflammasome EVs and TLR/UT EVs cluster separately from each other, with only very few exceptions. ns: not significant, *: *P‐*value < 0.05, **: *P‐*value < 0.01, ***: *P‐*value < 0.001, ****: *P‐*value < 0.0001

To analyse the transcriptomic content of 10K and SEC EVs released upon either inflammasome (EVs_inflammasome_) or TLR (EVs_TLR_) stimulation (Figures 2a, S3a) we performed gene‐expression profiling using a microarray with comprehensive coverage of the transcribed genome. Efficient priming of EV donor cells was confirmed by the TNFα released by cells after LPS priming or long‐term TLR stimulation with R848 or P3CSK4 (Figure 2b). Likewise, IL‐1β release (Figure 2c) and induction of cell death (Figure 2d) confirmed successful inflammasome activation. As expected, NLRP3 and NLRC4 activation, but not LPS priming alone, induced IL‐1β release and pyroptosis. In contrast, long‐term TLR stimulation (R848 and P3CSK4), but also short‐term TLR stimulation (LPS + R848), induced moderate IL‐1β release, while cell death levels remained low.

The samples separated well regarding their EV class (10K vs. SEC EVs) and the EV‐inducing stimulus (EVs_inflammasome_ vs. EVs_TLR/UT_) in a multidimensional scaling (MDS) plot (Figure S3b) and in a heat map of the most variable coding transcripts across all samples (Figure 2e).

**FIGURE 3:**
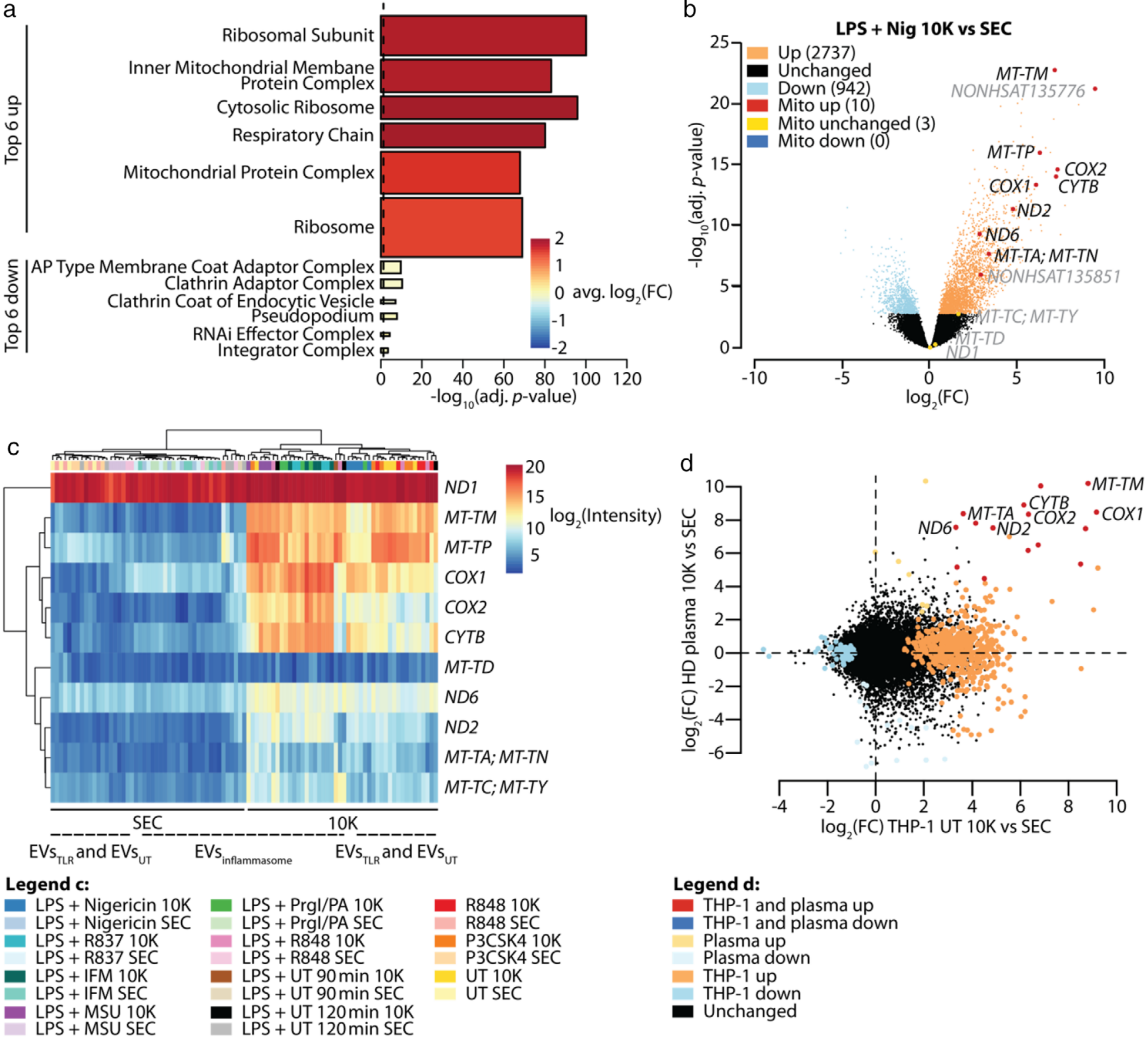
10K EVs are enriched in mitochondrially encoded transcripts compared to SEC EVs. (a), Gene set testing results depicted in Figure S3c were combined using the combined *P* value function from the EGSEA package. The top six up‐regulated and the top six down‐regulated cellular component GO terms were plotted from the combined 10K versus SEC comparison across all stimulation groups. Bars are coloured by average log_2_(FC) of the gene set across all comparisons. Bar width represents the number of genes in the respective gene set. Dashed line indicates adjusted *P*‐value threshold. (b), Volcano plot comparing log_2_(FC) and −log_10_(adjusted *P*‐value) of 10K versus SEC EVs released upon LPS + nigericin stimulation. Significantly up‐ (orange) or down‐regulated (light blue) as well as mitochondrially encoded transcripts (red, blue) are highlighted. Not significantly changed mitochondrially encoded transcripts (yellow) as well as mitochondrially encoded lncRNAs are labelled in grey. (c), Heat map depicting normalized log_2_ expression values of all detected mitochondrially encoded transcripts (except lncRNAs) across all samples. Multiple transcript labels per row indicate transcripts which cannot be distinguished from each other with the probe set used. Lines indicating samples derived from inflammasome EVs and TLR/UT EVs are dashed to indicate that sample clustering was not perfect, meaning that the majority of inflammasome EVs and TLR/UT EVs cluster separately from each other, with only very few exceptions. (d), Comparison of EVs released from untreated THP‐1 macrophages and plasma EVs. log_2_(FC) of transcripts comparing 10K versus SEC EVs released from unstimulated THP‐1 macrophages were plotted against log_2_(FC) of transcripts comparing 10K versus SEC EVs present in 2 ml of frozen plasma. Significant transcripts in each comparison are highlighted by colour. Mitochondrially encoded transcripts are labelled if significantly up‐regulated in THP‐1 cells and plasma. Adjusted *P‐*value threshold = 0.05. Mito = mitochondrially encoded, adj. = adjusted, avg. = average, FC = fold change

We noticed a considerable overlap in the consistently up‐regulated transcripts and gene sets comparing 10K versus SEC EVs across all stimuli, with some variation between EVs_inflammasome_ compared to EVs_TLR_ (Figure S3c). The top six up‐regulated cellular component GO terms in the combined 10K versus SEC comparison, including all stimuli (Figure 3a), revealed that mitochondrial and ribosomal gene sets were strongly over‐represented in 10K EVs compared to SEC EVs.

The identified enrichment of GO terms associated with mitochondria within 10K EVs prompted us to quantify mitochondrially encoded transcripts. Out of 13 such transcripts on the microarray, 10 were among the most differentially and significantly up‐regulated transcripts comparing 10K_LPS+Nig_ with SEC _LPS+Nig_ (Figure 3b). However, this was observed across all stimuli of interest, including 10K_inflammasome_, 10K_LPS_ and 10K_TLR_ (Figure 3c), indicating that the packaging of mitochondrially encoded transcripts within 10K EVs was not specific for EVs_NLRP3_. However, the enrichment of mitochondrially encoded transcripts within 10K EVs seems to be physiologically relevant since it was also detected when comparing 10K and SEC EVs isolated from blood plasma of healthy donors (Figure 3d).

To identify how EVs_inflammasome_ and EVs_TLR_ differed from each other, we performed a background correction against the priming only control (for LPS‐primed stimuli) or the untreated control (for long‐term TLR stimuli). Various transcripts were released in a significantly different manner compared to the background within EVs_inflammasome_ (Figure S3d). Interestingly, many transcripts present in 10K or SEC priming EVs were significantly less abundant in EVs_inflammasome_, meaning that these transcripts were depleted upon full stimulation (Figure S3d). Transcripts that are commonly changed within EVs_inflammasome_ could serve as biomarkers in inflammasome‐associated diseases. In contrast, only a few background‐corrected transcripts were associated with EVs_TLR_ (Figure S3d), indicating that the transcripts within EVs_TLR_ are not different from the transcripts within EVs_UT_.

**FIGURE 4:**
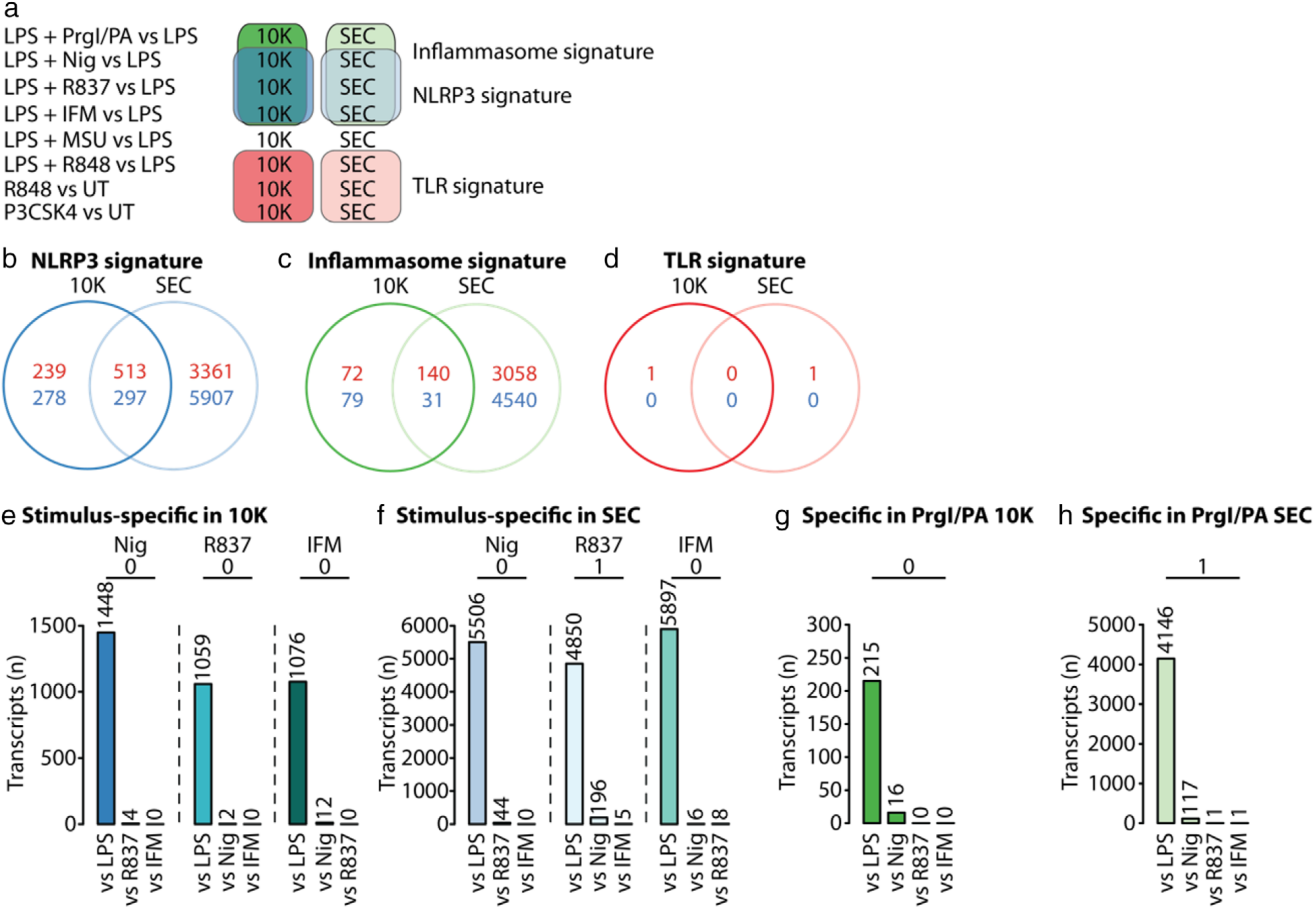
EV content released upon inflammasome stimulation is conserved. (a), Schematic diagram illustrating which stimuli contributed to which signature. (b–d), Venn diagrams visualizing the overlap between 10K and SEC EV content for transcripts in the NLRP3 signature (b), inflammasome signature (c), and TLR signature (d). (e, f), Number of transcripts that were specifically released in 10K (e) or SEC EVs (f) upon stimulation with the NLRP3 stimulus stated on top of the figure compared to the stimuli stated on the x‐axis. (g, h), Number of transcripts which were specifically released in 10K (g) or SEC EVs (h) upon stimulation with PrgI/PA compared to the stimuli stated on the x‐axis. Adjusted *P‐*value threshold = 0.05

There was a vast overlap in significantly up‐ or down‐regulated transcripts in 10K and SEC EVs_NLRP3_ compared to the priming only background (Figure S3e,f), with EVs_LPS+MSU_ as an exception (Figure S3g,h). Since the release of EVs_LPS+MSU_ was not NLRP3‐dependent (Figure S2k), we excluded EVs_LPS+MSU_ from further analysis. The overlap in consistently changed transcripts within EVs_NLRP3_ prompted us to define EV signature transcripts (Figures 4a–d and S4a): the NLRP3 signature contains all transcripts that were consistently changed in EVs_LPS+Nig_, EVs_LPS+R837_ and EVs_LPS+IFM_ compared to EVs_LPS_; the inflammasome signature contains all NLRP3 signature transcripts that were also consistently changed in EVs_LPS+PrgI_ compared to EVs_LPS_; and the TLR signature contains all transcripts that were consistently changed in EVs_LPS+R848_, EVs_R848_, and EVs_P3CSK4_ compared to EVs_LPS_ or EVs_UT_. All transcripts within the inflammasome signature were also part of the NLRP3 signature. The proportion of transcripts specifically detected in the NLRP3 signature, but not in the inflammasome signature, was limited (Figure S4a).

Of note, almost no transcripts were released in a stimulus‐specific manner within EVs_NLRP3_ (Figure 4e,f; one exception in Figure S4b) or when comparing EVs_LPS+PrgI_ to EVs_NLRP3_ (Figure 4g,h; one exception in Figure S4c). The general lack of stimulus‐specific transcripts indicates that the content of EVs released upon inflammasome stimulation was not determined by sensing of the inflammasome‐activating stimulus itself but likely through a mechanism that is mediated further downstream of inflammasome activation.

To investigate the differences in NLRP3 signature transcripts between 10K and SEC EVs, a gene set over‐representation analysis on transcripts significantly up‐regulated within either the 10K or SEC NLRP3 signature was performed (Figure S4d). While mitochondrial and ribosomal gene sets featured highly in background‐corrected 10K and SEC EVs, ER‐ or cytoskeleton‐associated gene sets were uniquely enriched within 10K or SEC EVs_NLRP3_, respectively (Figure S4d). These data imply that transcripts are sorted towards a specific EV class in response to particular triggers. A classification of NLRP3 signature transcripts according to their biological functionality showed a similar distribution of transcript types in 10K and SEC EVs within up‐regulated (Figure S4e) and down‐regulated (Figure S4f) NLRP3 signature transcripts. However, there was a large difference in transcript types between NLRP3 signature transcripts that were up‐ versus down‐regulated (Figure S4e vs. f), suggesting that sorting of transcripts into EVs released upon different stimulations may be regulated by the transcript type. In contrast to the NLRP3 and inflammasome signatures, almost no transcripts were significantly changed in all EVs_TLR_ compared to their respective background control (Figure 4d; *CCL20* within 10K_TLR_ and *IL1B* within SEC_TLR_).

### 3.4 Inflammasome EVs induce ISGs in EV recipient macrophages

**FIGURE 5:**
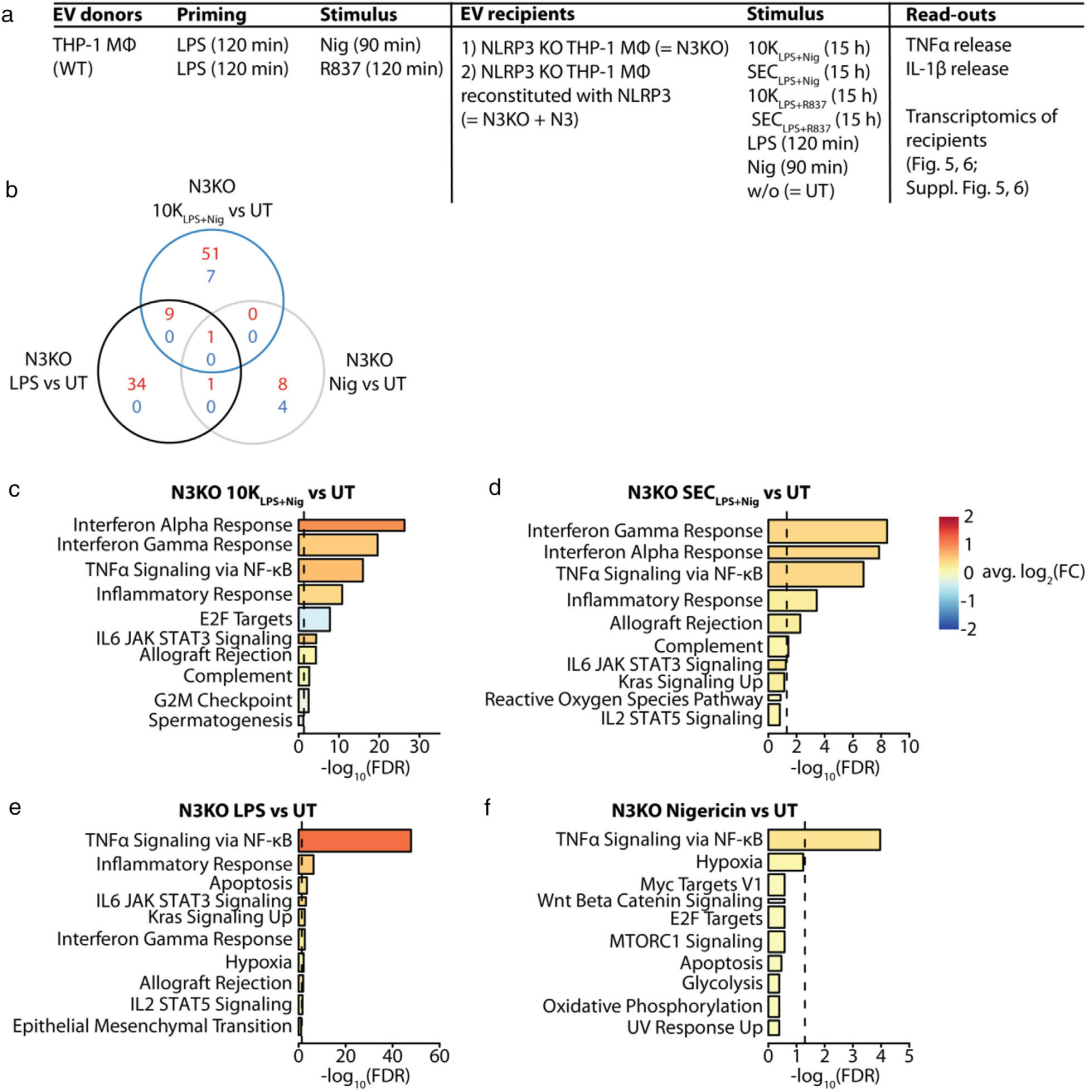
Inflammasome EVs and EV‐inducing stimuli have different effects on gene expression in recipient cells. (a), Table summarizing cells, their stimulation and the read‐outs performed in experiments depicted in (b–f), Figures 6, S5 and S6. MΦ: macrophage. EV release by PMA‐differentiated WT THP‐1 macrophages was induced by stimulation with LPS + nigericin (10K_LPS+Nig_ and SEC_LPS+Nig_ EVs) or with LPS + R837 (10K_LPS+R837_ and SEC_LPS+R837_ EVs). EVs were transferred at a 40:1 EV donor cell to EV recipient cell ratio to 3.25 · 10^5^ PMA‐differentiated THP‐1 recipient macrophages per condition for 15 h. Recipient cells were either NLRP3 KO (N3KO) THP‐1 macrophages or NLRP3‐reconstituted NLRP3 KO (N3KO + N3) THP‐1 macrophages. As controls, recipient cells were stimulated with 200 ng/ml LPS, 10 μM nigericin or 20 μg/ml R837, or left untreated. Cellular RNA (from recipient cells) as well as vesicular RNA (from EVs released by EV donor cells) was extracted and subjected to 3′ sequencing. (b), Venn diagram visualizing the overlap in up‐ (red) or down‐regulated transcripts (blue) comparing N3KO recipients treated with 10K_LPS+Nig_ EVs, N3KO recipients treated with LPS and N3KO recipients treated with nigericin. (c–f), Gene set testing was performed using the camera function from the limma package. Plots show the top 10 hallmark gene sets in the comparison indicated: N3KO recipients treated with 10K_LPS+Nig_ EVs versus untreated (c), N3KO recipients treated with SEC_LPS+Nig_ EVs versus untreated (d), N3KO recipients treated with LPS versus untreated (e), and N3KO recipients treated with nigericin versus untreated (f). Bar colour indicates the average log_2_(FC) of all genes within a gene set for the comparison of interest. Bar width represents the number of genes within a gene set. Dashed line indicates adjusted *P‐*value threshold = 0.05. FDR = false discovery rate‐adjusted *P*‐value

After characterizing the transcriptional content of EVs_NLRP3_ in detail, we investigated the functional effects of such EVs on macrophages. Since the content of EVs_inflammasome_ was conserved regardless of the inflammasome activator (Figure 4), only transcriptional changes induced by EVs_LPS+Nig_ and EVs_LPS+R837_ were assessed (Figure 5a). To distinguish between NLRP3‐dependent and ‐independent effects, we transferred EVs to NLRP3 KO THP‐1 macrophages (N3KO THP‐1) and NLRP3 KO THP‐1 macrophages that had been reconstituted with low levels of NLRP3 (N3KO+N3 THP‐1) (Figure 5a). Importantly, EVs were free of endotoxin (Figure S5a), despite the LPS priming of EV donor cells. We assessed the priming levels and NLRP3 activation of recipient cells by measuring TNFα (Figure S5b) and IL‐1β (Figure S5c) levels in the supernatant. Notably, the recipient cells exposed to 10K_LPS+Nig_ or 10K_LPS+R837_ (Figure S5c) did not release IL‐1β, yet IL‐1β was present within the transferred EVs themselves (Figure S5d).

While co‐incubation of SEC_NLRP3_ with recipient macrophages did not induce any significant individual changes in expression (data not shown), 10K_LPS+Nig_ caused transcriptional changes in recipient cells that differed from the effects of the EV‐inducing stimuli themselves (i.e., LPS or nigericin) (Figure 5b). Gene set testing on responses seen in N3KO THP‐1 macrophages treated with EVs_LPS+Nig_ or EVs_LPS+R837_ showed that interferon (IFN) response gene sets ranked highly (Figure 5c,d, R837 data not shown for conciseness), which was not the case when N3KO THP‐1 macrophages were instead stimulated with LPS or nigericin (Figure 5e,f).

Agreeing with a similar transcriptional content of EVs_inflammasome_, the most significantly differentially expressed genes upon 10K_LPS+Nig_ and 10K_LPS+R837_ transfer showed similar fold changes in recipient macrophages (Figure S5e).

**FIGURE 6:**
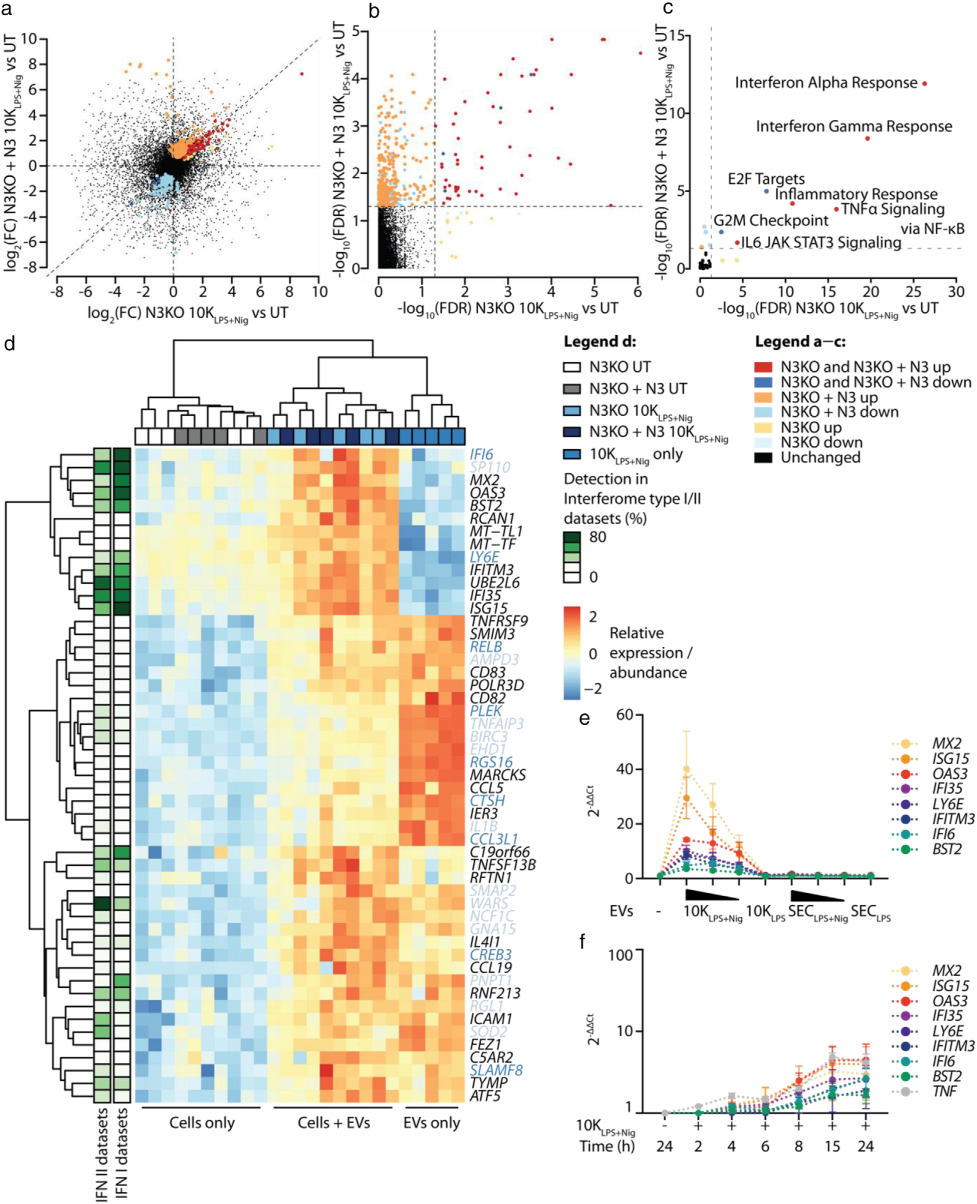
Inflammasome EVs induce an IFN signature independently of NLRP3 expression in recipient cells. (a, b), Comparison of 10K_LPS+Nig_ EV effect on N3KO recipient cells and N3KO + N3 recipient cells. Either log_2_(FC) N3KO 10K_LPS+Nig_ versus untreated (UT) was plotted against log_2_(FC) N3KO + N3 10K_LPS+Nig_ versus untreated (a) or −log_10_(adjusted *P‐*value) N3KO 10K_LPS+Nig_ versus untreated was plotted against −log_10_(adjusted *P‐*value) N3KO + N3 10K_LPS+Nig_ versus untreated (b). FDR = false discovery rate‐adjusted *P*‐value. Significantly different transcripts are highlighted by colour. (c), Gene set testing was performed using the camera function from the limma package. Plot shows the −log_10_(adjusted *P‐*value) of the hallmark gene sets that were significantly different upon 10K_LPS+Nig_ transfer in N3KO or N3KO + N3 recipient cells. (d), Heat map depicting transcripts that were significantly up‐regulated upon 10K_LPS+Nig_ transfer in N3KO as well as N3KO + N3 recipient cells. Left legend indicates in what percentage of Interferome data sets (type I or type II) the respective transcript was identified. Blue labelling of transcripts indicates them as part of the 10K (dark blue) or SEC (light blue) NLRP3 signature (as defined in Figure 4). Adjusted *P‐*value threshold = 0.05. (e), EV release by PMA‐differentiated WT THP‐1 macrophages was induced by stimulation with LPS + nigericin (10K_LPS+Nig_ and SEC_LPS+Nig_) or LPS alone (10K_LPS_ and SEC_LPS_). 10K_LPS+Nig_ and SEC_LPS+Nig_ EVs were transferred at a 40:1, 20:1, and 10:1 EV donor cells to EV recipient cell ratio and 10K_LPS_ and SEC_LPS_ EVs were transferred at a 40:1 EV donor cell to EV recipient cell ratio to 3.25 · 10^5^ PMA‐differentiated WT THP‐1 recipient macrophages per condition for 15 h. RNA was isolated from EV recipient cells and subjected to qRT‐PCR. Pooled data of *n* = 3, each in technical duplicates, mean ± SEM. f, 10K_LPS+Nig_ EVs were transferred at a 40:1 EV donor cell to EV recipient cell ratio to 3.25 · 10^5^ PMA‐differentiated WT THP‐1 recipient macrophages per condition for the period of time indicated. RNA was isolated from EV recipient cells and subjected to qRT‐PCR. Pooled data of *n* = 3, each in technical duplicates, mean ± SEM

Characterization of the effect of 10K_LPS+Nig_ on N3KO THP‐1 macrophages versus N3KO+N3 THP‐1 macrophages showed that the most significant differentially expressed genes had similar fold changes in both recipient cell types (Figure 6a). Additionally, most of the significantly changed genes upon 10K_LPS+Nig_ transfer in N3KO THP‐1 macrophages also changed significantly upon 10K_LPS+Nig_ transfer in N3KO+N3 THP‐1 macrophages (Figure 6b). In both recipient cell lines, the IFN response gene sets were the most predominantly enriched (Figure 6c).

The genes that were significantly up‐regulated upon 10K_LPS+Nig_ transfer in both recipient cell types clustered clearly by the recipient cell stimulation (untreated cells, EV recipient cells and EVs only; Figure 6d). Genes that were lowly expressed in cells and not abundant in EVs but significantly increased in cells upon EV transfer were likely to be induced as a response to EV uptake rather than simply transferred via EVs to cells. Most of these genes (upper cluster in Figure 6d) were also frequently up‐regulated in type I or type II IFN data sets of the Interferome, a database for ISGs. Intriguingly, half of the highly abundant genes in EVs were previously identified within the NLRP3 signature (blue genes in Figure 6d). The induction of ISGs by 10K_LPS+Nig_ but not 10K_LPS_, SEC_LPS+Nig_ or SEC_LPS_ was confirmed to be EV dose‐dependent and temporally correlated with EV uptake by qRT‐PCR (Figure 6e,f). Additionally, 10K_LPS+Nig_ also induced ISGs in primary human monocyte‐derived macrophages (hMDMs) (Figure S7a,b).

To identify a common regulator of the genes induced by 10K_LPS+Nig_, we performed transcription factor binding site (TFBS) prediction within the promoters. In line with the induction of ISGs upon EV transfer, STATs, IRFs, and NF‐κB transcription factors (TFs) ranked highly within 10K_LPS+Nig_‐treated N3KO and N3KO+N3 recipient macrophages (Figure S6a,c). In contrast, TFBS prediction in promoters of genes that were solely significantly different upon 10K_LPS+Nig_ EV transfer in N3KO+N3 THP‐1 macrophages but not in N3KO THP‐1 macrophages yielded only minor and not consistently significant TF enrichment (Figure S6b,d). Enrichment of NF‐κB TFs but not IRFs within promotors of up‐regulated genes upon LPS treatment confirmed that LPS treatment led to a different TF profile than EV treatment (Figure S6e,f).

In summary, we observed that EVs_NLRP3_ induced an IFN signature in recipient cells, which was not induced by the EV‐inducing NLRP3 activators themselves and did not require NLRP3 in EV recipient cells.

### 3.5 Inflammasome EVs contain IFNβ responsible for the induction of ISGs in EV recipient cells

**FIGURE 7:**
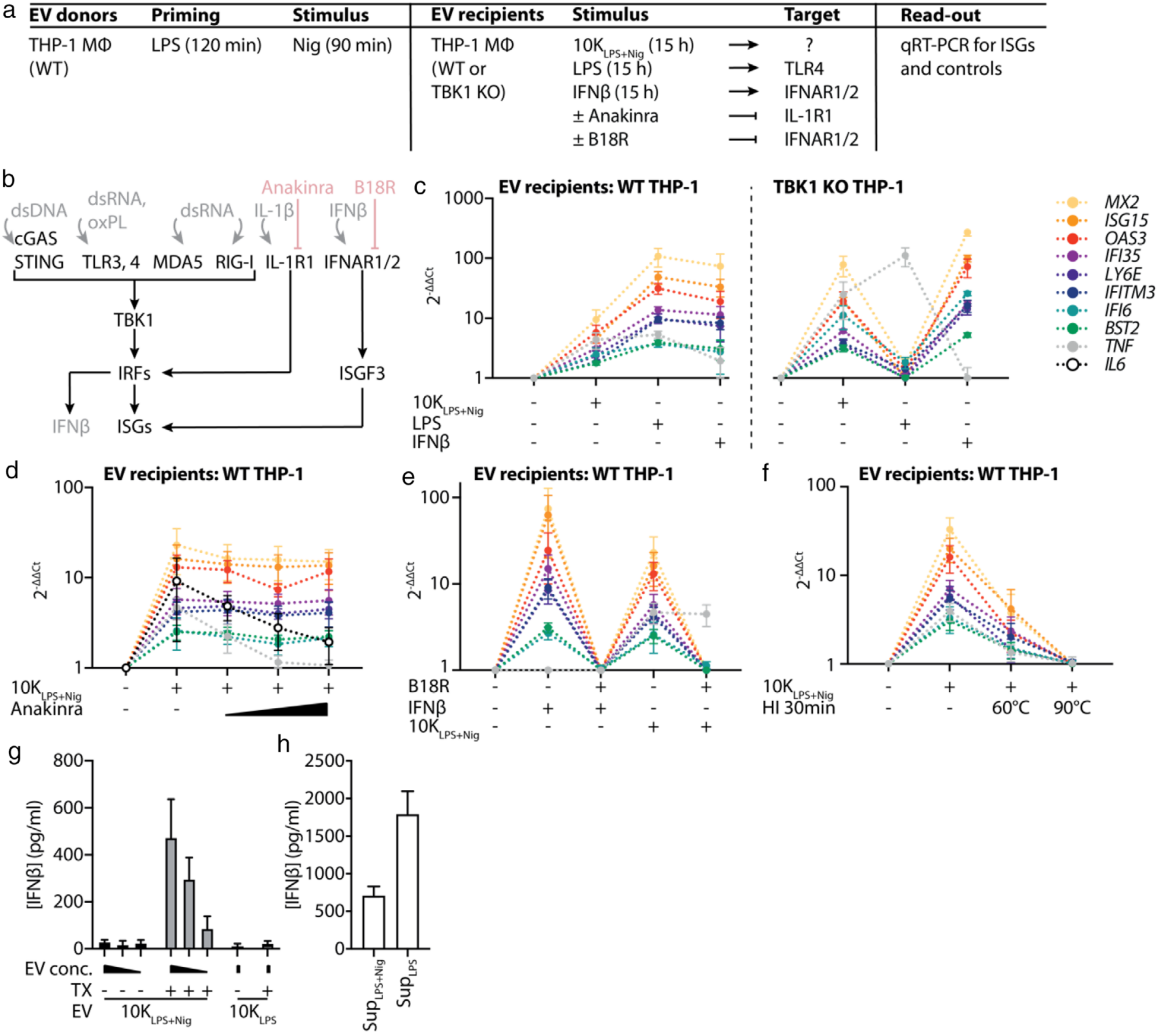
NLRP3‐induced EVs contain IFNβ protein that is responsible for the induction of ISGs in EV recipient cells. (a), Table summarizing cells, their stimulation and the read‐outs performed in experiments depicted in (c–f). MΦ: macrophage. (b), Schematic diagram of signalling pathways leading to ISG expression. Ligands are displayed in grey. Compounds interfering with receptor signalling are displayed in red. Anakinra is a recombinant IL‐1 receptor (IL‐1R1) antagonist competing with endogenous IL‐1β for receptor binding. B18R is a type I IFN decoy receptor competing with endogenous IFNβ for receptor binding. cGAS: cyclic GMP‐AMP synthase, IFNAR1/2: IFNα/β receptor, IRF: IFN regulatory factor, ISGF3: IFN‐stimulated gene factor 3, oxPL: oxidized phospholipid, RIG‐I: retinoic acid‐inducible gene I, STING: stimulator of IFN genes, TBK1: TANK‐binding kinase 1. c, EV release by PMA‐differentiated WT THP‐1 macrophages was induced by stimulation with LPS + nigericin (10K_LPS+Nig_). Per condition, 3.25 · 10^5^ PMA‐differentiated WT THP‐1 macrophages (left) or TBK1 KO THP‐1 macrophages (right) were stimulated with 10K_LPS+Nig_ EVs (40:1 EV donor cell to recipient cell ratio), 200 ng/ml LPS, or 5 · 10^3^ U/ml IFNβ for 15 h. RNA was isolated from recipient cells and subjected to qRT‐PCR. (d), Per condition, 3.25 · 10^5^ PMA‐differentiated WT THP‐1 macrophages were stimulated with 10K_LPS+Nig_ EVs (40:1 EV donor cell to recipient cell ratio) in the absence or presence of Anakinra (0.05 μg/ml, 0.5 μg/ml, 5 μg/ml). RNA was isolated from recipient cells and subjected to qRT‐PCR. (e), Per condition, 3.25 · 10^5^ PMA‐differentiated WT THP‐1 macrophages were incubated in the absence or presence of 5 μg/ml B18R for 1 h and subsequently stimulated with 5 · 10^3^ U/ml IFNβ or 10K_LPS+Nig_ EVs (40:1 EV donor cell to recipient cell ratio) for 15 h. RNA was isolated from recipient cells and subjected to qRT‐PCR. f, 10K_LPS+Nig_ EVs were incubated on ice or at 60°C or 90°C for 30 min. Subsequently, 3.25 · 10^5^ PMA‐differentiated WT THP‐1 macrophages per condition were stimulated with 10K_LPS+Nig_ EVs (40:1 EV donor cell to recipient cell ratio) for 15 h. RNA was isolated from recipient cells and subjected to qRT‐PCR. HI = heat inactivation. (g, h), EV release by PMA‐differentiated WT THP‐1 macrophages was induced by stimulation with LPS + nigericin (10K_LPS+Nig_) or LPS alone (10K_LPS_). 10K_LPS+Nig_ EVs released by 6.5, 4, and 2 · 10^6^ THP‐1 macrophages and 10K_LPS_ EVs released by 6.5 · 10^6^ THP‐1 macrophages were incubated in the presence or absence of 0.1% Triton X‐100 (TX) and subsequently subjected to IFNβ HTRF (g). Supernatants (sups) of LPS + nigericin or LPS only stimulated cells obtained after 10,000 g spin were concentrated until they had the same volume EVs in (g) were resuspended in (and thereby sups and EVs corresponded to what was released from 6.5 · 10^6^ THP‐1 macrophages) and subsequently subjected to IFNβ HTRF (h). Pooled data of *n* = 3, each in technical duplicates, mean ± SEM (c−f) or technical triplicates (g, h), mean + SEM

Next, we aimed at identifying how 10K_LPS+Nig_ induced ISGs in recipient macrophages (Figure 7a). Generally, ISG induction is downstream of multiple pattern recognition receptors (PRRs) or cytokine receptors that could be engaged by various receptor ligands on or within EVs (Figure 7b). Engagement of cGAS, TLRs, MDA5 or RIG‐I by EVs could be ruled out since 10K_LPS+Nig_ were still capable of inducing ISGs in TBK1 KO THP‐1 macrophages (Figure 7c). Accordingly, the STING inhibitor H‐151 had no effect on EV‐evoked ISG induction (Figure S7a,c) and murine 10K_LPS+Nig_ EVs were still capable of inducing ISGs in STING KO, cGAS KO, and UNC93B KO bone marrow‐derived macrophages (BMDMs; Figure S7a,d). Additionally, vesicular IL‐1β protein was not responsible for ISG induction via IL‐1R1 since increasing doses of the soluble IL‐1 receptor antagonist anakinra only abolished *TNF* and *IL6* expression, but not ISG induction (Figure 7d).

In contrast, the soluble decoy type I IFN receptor B18R completely blocked 10K_LPS+Nig_‑mediated ISG induction, but not that of *TNF* (Figure 7e). Since these data suggested that EVs provide a source of IFNβ protein, we heat‐inactivated EVs before transfer. Indeed, ISG induction was heat‐sensitive and, therefore, most likely evoked by a protein (Figure 7f). Our hypothesis was confirmed by the detection of IFNβ in lysed, but not intact, EVs (Figure 7g), indicating that IFNβ is present within and not solely attached to 10K_LPS+Nig_. Besides IFNβ contained within 10K_LPS+Nig_, soluble IFNβ was additionally detected within the supernatant, as expected upon TLR4 stimulation with LPS (Figure 7h). Notably, the overall amount of IFNβ released (i.e., in 10K EVs and supernatant combined) was roughly equivalent upon LPS and LPS + nigericin stimulation (Figure 7g,h); however, only LPS + nigericin stimulation induced its packaging into EVs.

Since IFNβ is known to act species‐specifically, we were not surprised to see that human 10K_LPS+Nig_ were solely capable of inducing ISGs in human but not murine recipient macrophages (Figure S7e). Equivalently, murine 10K_LPS+Nig_ EVs did not induce ISGs in human recipient cells (Figure S7e).

### 3.6 Inflammasome EVs dampen NLRP3 activation in un‐primed recipient cells

**FIGURE 8:**
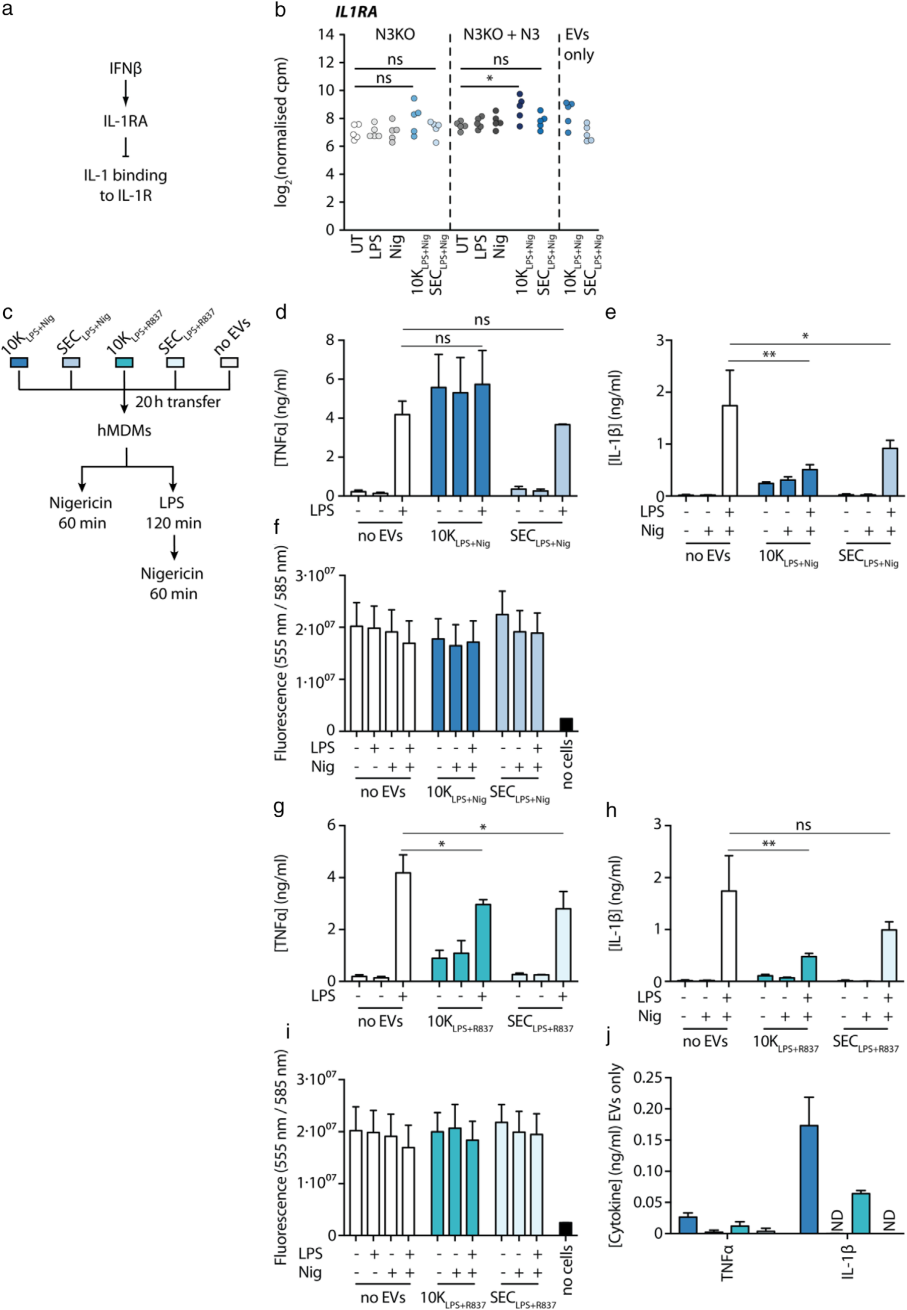
Inflammasome EVs dampen NLRP3 activation in un‐primed recipient cells. (a), Mechanism how IFNβ can interfere with inflammasome responses. (b), Expression levels of IL‐1 receptor antagonist (*IL1RA*) in differently treated N3KO and N3KO + N3 THP‐1 macrophages as well as transcript abundance of *IL1RA* in EVs only. (c), Schematic diagram of the experiment. PMA‐differentiated WT THP‐1 macrophages were stimulated with LPS + nigericin (10K_LPS+Nig_ and SEC_LPS+Nig_) or LPS + R837 (10K_LPS+R837_ and SEC_LPS+R837_) to induce EV release. 8 · 10^4^ primary human monocyte‐derived macrophages (hMDMs) per condition were co‐cultured with those EVs for 20 h at a 40:1 EV donor cell to EV recipient cell ratio or left untreated. Afterwards, recipient cells were stimulated with LPS + nigericin or nigericin only. (d, g), Tissue culture supernatants were taken after EV transfer (10K_LPS+Nig_ and SEC_LPS+Nig_ in (d), 10K_LPS+R837_ and SEC_LPS+R837_ in (g)) and priming and subjected to TNFα HTRF. (e, h), Tissue culture supernatants were taken after full stimulation (10K_LPS+Nig_ and SEC_LPS+Nig_ in (e), 10K_LPS+R837_ and SEC_LPS+R837_ in (h)) and subjected to IL‐1β HTRF. (f, i), After full stimulation (10K_LPS+Nig_ and SEC_LPS+Nig_ in (f), 10K_LPS+R837_ and SEC_LPS+R837_ in (i)), cell death levels were determined by performing CTB assays on recipient cells. (j), Cytokine levels in EVs only were determined by TNFα and IL‐1β HTRF in the same volume cells were stimulated in. Pooled data of *n* = 3, each in technical triplicates, mean + SEM. ns: not significant, *: *P‐*value < 0.05, **: *P‐*value < 0.01, ND = not detected

IFNβ is known to negatively interfere with inflammasome responses, for example, by inducing the endogenous IL‐1 receptor antagonist (IL‐1RA; Figure 8a) (Huang et al., 1995; Tilg et al., 1993). Since we found that 10K_LPS+Nig_ contained IFNβ protein and indeed *IL1RA* was up‐regulated upon 10K_LPS+Nig_ transfer (Figure 8b), we hypothesized that EVs_NLRP3_ might affect inflammasome responses in EV recipient macrophages. To test this, we treated hMDMs with EVs_LPS+Nig_ and EVs_LPS+R837_ and subsequently stimulated the EV recipient cells with LPS and nigericin (Figure 8c). There was no significant difference in priming efficiency observed between LPS‐treated and EV_LPS+Nig_‐treated cells (Figure 8d), although priming efficiency was slightly decreased upon EV_LPS+R837_ treatment (Figure 8g). Upon subsequent nigericin stimulation, IL‐1β release after 10K_LPS+Nig_, SEC_LPS+Nig_, and 10K_LPS+R837_ transfer was significantly lower in EV recipient cells than in non‐recipient cells (Figure 8e,h), meaning that EVs_NLRP3_ dampened inflammasome responses in un‐primed EV recipient cells. This was not due to cell viability differences between EV recipient and non‐recipient cells (Figure 8f,i). The reduction of IL‐1β released was even more striking, considering that EVs_NLRP3_ themselves contain and therefore transfer small amounts of IL‐1β to recipient cells (Figure 8j).

When investigating if EVs can serve as an inflammasome activator after initial LPS priming of hMDMs (Figure S8a), we saw that especially 10K_LPS+Nig_ prompted substantial IL‐1β release compared to the priming only condition (Figure S8b), which was not consistently accompanied by an increase in cell death (Figure S8c). This induced IL‐1β release from primed EV recipient cells could not be solely explained by transferred IL‐1β protein within 10K EVs (Figure S8d).

In summary, 10K_LPS+Nig_ augmented inflammasome responses when transferred to pre‐primed macrophages. However, when 10K_LPS+Nig_ or 10K_LPS+R837_ were transferred to un‐primed primary macrophages, subsequent inflammasome activation was inhibited. These data demonstrate that inflammasome‐triggered EVs can modulate bystander cells' responsiveness and suggest they are part of a negative feedback loop.

## 4 DISCUSSION

Only centuries ago, infections represented the primary cause of death in children and adults alike. The evolutionary constraints, under which humans' selection has occurred, have thus favoured selecting genes that provide sensitive and effective anti‐microbial immune responses. It is likely that antagonistically pleiotropic genes, such as some members of the inflammasome sensors, have been selected for, as they convey a benefit for infection control in early life that outweighs their detrimental influence in the post‐reproductive period of life. Some inflammasome sensors, most prominently NLRP3, have been identified as drivers of chronic inflammatory pathologies that affect many patients in our aging societies. Basic research has uncovered many of the activation mechanisms for the NLRP3 inflammasome and identified the instigators of NLRP3 pathway activity, such as crystals or aggregated substances. Nevertheless, analysing the triggers of inflammasome activation *in vivo* remains a challenge but could help select patients expected to benefit from a specific inflammasome‐directed therapy.

Measuring inflammasome‐dependent cytokines as an indicator of inflammasome activity has, however, thus far been a challenge. Steady‐state measurements of immune activity, such as assessing the cytokine levels in body fluids, are possible but have limitations. Large inter‐individual and day‐to‐day variability of cytokine levels has so far hindered the definition of internationally accepted standard values for cytokines, chemokines, or other mediators related to immune cell activation in humans. Instead, other markers induced by inflammatory cytokines, such as acute‐phase reactants, are established to measure more general immune activation. For example, C‐reactive protein (CRP) is an established parameter to assess inflammation or the risk of coronary events in atherosclerosis. It has been used as a stratifier in large clinical trials testing the effectiveness of anti‐inflammatory therapies in atherosclerosis (e.g., the CANTOS trial (Ridker et al., 2017)). Hence, other pathway activity indicators, such as EVs, could represent more reliable and specific inflammasome activation markers. Therefore, the first purpose of this study was to characterize the content of EVs_inflammasome_ thoroughly.

To our knowledge, this is the first study profiling different EV classes and their RNA content released upon stimulation with various inflammasome activators. We showed that pathways eliciting caspase 1‐driven cell death induced EV release, while stimuli that do not elicit pyroptosis were weaker stimuli for EV release. However, NLRC4 stimulation caused the release of all EV classes, while only the release of 2K_LPS+PrgI_ and 10K_LPS+PrgI_ was partially gasdermin D‐dependent, which suggests that the release of EVs_inflammasome_ is not solely a passive, pyroptosis‐driven event, but is instead highly regulated.

We saw that 10K and SEC EVs released from the same EV donor cells differed considerably from each other in their RNA content. This strongly suggests the existence of a regulated RNA sorting mechanism and argues against a stochastic release of material upon cell rupture and pyroptosis. One main difference identified between 10K and SEC EVs was the strong enrichment of mitochondrially encoded transcripts in 10K EVs. Mitochondria, which themselves pellet at 8000–10,000 g (Wieckowski & Wojtczak, 2015), could be passively released during pyroptosis and contaminate isolated EVs. However, this potential technical artefact is rather unlikely since mitochondrial proteins (Bernimoulin et al., 2009), DNA (Garcia‐Martinez et al., 2016), and even entire mitochondria (Hough et al., 2018) were reported as EV content in experimental settings not including cell death. Furthermore, in our experiments, 10K_LPS_ and 10K_UT_ were enriched in mitochondrially encoded transcripts, even though their donor cells did not die. Finally, blood plasma, which has been shown to contain cell‐free mitochondria in several pathologies (Ellinger et al., 2008; Sudakov et al., 2015) but not in healthy individuals, contained 10K EVs enriched in mitochondrially encoded transcripts, demonstrating *in vivo* relevance. However, these data do not exclude an active release mechanism for mitochondria. Indeed, extracellular mitochondria are considered a neglected subtype of EVs actively released from monocytes upon LPS stimulation (Puhm et al., 2019). Furthermore, recent work shows that gasdermin D and E can perforate mitochondria, which results in cytochrome C release, a potent signal inducing apoptosis (Rogers et al., 2019). Pores within mitochondria induced upon NLRP3 activation could allow for the release of intra‐mitochondrial components and their packaging into EVs. Since there currently is a lack of markers that can clearly distinguish ectosomes from exosomes (Choi et al., 2013; Kalra et al., 2013), we suggest that mitochondrially encoded transcripts can serve this purpose.

We defined an EV‐associated NLRP3 signature by the transcripts that we found commonly in EVs released upon stimulation with nigericin, R837, and IFM. Likewise, we described an EV‐associated inflammasome signature as the overlap between NLRP3 signature transcripts and transcripts released upon NLRC4 stimulation. The large number of inflammasome signature transcripts suggests that the release of EVs_inflammasome_ and their cargo sorting are downstream of caspase 1. In contrast, there were only very few transcripts significantly different in EVs_TLR_ compared to EVs_UT_. In light of TLR stimulation inducing substantial transcriptomic changes in cells, this lack of a common EV‐associated TLR signature is particularly compelling and highlights the relevance of the inflammasome signature's specificity. The inflammasome and NLRP3 signatures may provide EV‐associated biomarkers for pathologies involving inflammasome activation.

It is of translational relevance to understand the upstream instigators of inflammation and unravel the downstream effectors of these pathways. How inflammasome‐mediated cell death and EVs are involved in the inflammatory response remains ill understood. Whereas in humans with CAPS, anti‐IL‐1β therapy has proved efficacious and can mitigate most of the inflammatory symptoms (Booshehri & Hoffman, 2019), the situation in complex diseases, in which the appearance of triggers activates NLRP3, is likely different. In CAPS, NLRP3 pathway activity occurs after the mutated, and thereby overly active, NLRP3 protein is induced. Since IL‐1β drives NLRP3 gene induction, anti‐IL‐1β therapies in humans can stop a positive feedback loop that maintains most of the CAPS patients' inflammatory symptoms. However, in chronic inflammatory diseases, in which NLRP3 contributes to the pathogenesis, the appearance of a trigger for NLRP3 together with a priming event, which is not necessarily IL‐1β, can lead to chronic inflammasome activation. The relevance of inflammasome‐mediated pyroptosis and the related release of EVs into the environment is undoubtedly recognized by bystander cells. This is likely relevant for the pathophysiology of inflammation. Therefore, the second purpose of this study was to characterize the functional effects of EVs_NLRP3_ on bystander macrophages.

To dissect how the environment senses the macrophages' inflammasome‐driven suicide, we performed an unbiased systems analysis of how cells respond to EVs released from inflammasome‐triggered macrophages. To our knowledge, this is the first study broadly investigating the impact of EVs_NLRP3_ on the transcriptome of recipient cells. One study investigated small EVs released upon LPS and nigericin stimulation and claimed them to induce NF‐κB signalling, because NLRP3, pro‐IL‐1β, IL‐6, and TNFα were up‐regulated in recipient cells (Zhang et al., 2017). Although these proteins were either induced in recipients or transferred by EVs (Zhang et al., 2017), our data confirmed the TNFα signalling via NF‐κB gene set to be enriched upon EV_LPS+Nig_ transfer. Since that gene set was also strongly enriched in 10K_LPS+Nig_ themselves (data not shown) and they contained the NF‐κB‐driven proteins IL‐1β and TNFα, this is likely due to the transfer of components downstream of NF‐κB within EVs rather than an induction of NF‐κB itself in recipient cells.

However, the enrichment of interferon response gene sets was more prominent in our study. Although evoked by different mechanisms than the one presented here, EVs can induce IFN signatures in recipient cells. The ISGs *OAS2*, *MX1*, and *IFIT1* were induced in endothelial cells in response to vesicular, ROS‐elicited modifications in mitochondrial RNA, released upon long‐term LPS stimulation by THP‐1 monocytes (Puhm et al., 2019). Additionally, virus‐like particles devoid of viral proteins and genomic material, and even liposomes, can induce the expression of ISGs in a STING‐dependent but TLR‐ and RLR‐independent way (Holm et al., 2012). In contrast, we identified that EVs_NLRP3_ contain IFNβ, which, upon EV uptake, induces ISGs in recipient cells through the IFNα/β receptor (IFNAR). Since the EV‐contained IFNβ is protected from protease degradation, it can convey its effects to more distant cells than soluble IFNβ alone.

An open question concerning our model is the topology of IFNAR binding. IFNs are primarily described as soluble, secreted proteins. However, they were also previously identified as EV content within Vesiclepedia, a database of EV proteins and transcripts (Kalra et al., 2012). Extracellular IFNβ is considered to bind IFNAR (consisting of the two subunits IFNAR1 and IFNAR2) at the plasma membrane, which initiates ISG‐inducing signalling cascades (Lazear et al., 2019). Subsequently, IFNAR localizes to the endosome, which has been crucial for receptor signalling and its termination (Chmiest et al., 2016; Marchetti et al., 2006). Macrophages primarily take up EVs via phagocytosis (Feng et al., 2010), meaning that the EV cargo, such as IFNβ, is released into the endosome. Even though IFNAR can signal from the endosome (Altman et al., 2020), it remains to be elucidated if this can occur in the absence of receptor engagement at the plasma membrane.

Given our data that inflammasome activation can result in an IFN signature expression in bystander cells, we wondered whether CAPS patients, in which the NLRP3 pathway is overly active, display an IFN signature *in vivo*. CAPS is grouped into a disease spectrum of increasing severity in systemic inflammation: familial cold auto‐inflammatory syndrome (FCAS), Muckle‐Wells syndrome (MWS), and neonatal‐onset multisystem inflammatory disease (NOMID) (Aksentijevich et al., 2007). Up‐regulated IFNs or ISGs are not among the predominant elevated inflammatory blood markers, such as CRP, serum amyloid A, or IL‐6 (Booshehri & Hoffman, 2019). However, out of 31 IFN response‐specific genes significantly induced in pathologies with chronic elevation of IRGs, five genes (*PLSCR1*, *CD274*, *SAMD9*, *SOCS1*, and *IFIT5*) were also identified to be up‐regulated in NOMID patients compared to healthy controls (Kim et al., 2018). This demonstrates that, while CAPS in general and NOMID, in particular, are not primarily IFN‐driven, disease pathology is indeed characterized by the up‐regulation of some ISGs. CAPS patients typically experience re‐occurring inflammatory episodes. In light of IFNs having been shown to negatively regulate inflammasome responses, it is intriguing to speculate that the flares in CAPS patients may be endogenously negatively regulated by the release of EVs_NLRP3_.

We showed that EVs_NLRP3_ could either augment or dampen inflammatory responses depending on the recipient cells' priming state. Since EVs_NLRP3_ can circulate between the site of inflammation and the periphery, they could be involved in propagating inflammatory responses at the site of infection and the inhibition and termination of overshooting inflammation in the periphery, which are both required to maintain homeostasis. Future studies should translate these findings into clinical practice, where reliable biomarkers for inflammasome activation are needed to understand the contributions of inflammasomes to various pathologies. The analysis of different bystander cell types’ responses to inflammasome‐mediated EVs would, expectedly, reveal novel mechanisms that could explain how chronic inflammation is linked to the development of fibrosis, cancer, or organ dysfunction.

## AUTHOR CONTRIBUTIONS

Christina F. Budden designed the study, performed experiments, analysed and interpreted data, and wrote the manuscript. Linden J. Gearing analysed and interpreted data, gave experimental advice, and proofread the manuscript. Romina Kaiser and Lena Standke performed experiments and analysed data. Paul J. Hertzog supervised the study. Eicke Latz designed and supervised the study and wrote the manuscript.

## CONFLICT OF INTEREST

Eicke Latz is co‐founder and consultant of IFM Therapeutics. The remaining authors declare no competing interests.

## Supporting information



Supplementary Fig. 1: EV release upon NLRP3 activation with LPS + R837 temporally correlates with IL‐1β release and is an NLRP3‐, caspase 1‐, and gasdermin D‐dependent event. (a), Table summarizing cells, their stimulation and the read‐outs performed in experiments depicted in b−n. MΦ: macrophage. (b–l), 10 · 10^6^ PMA‐differentiated WT THP‐1 macrophages were primed with 200 ng/ml LPS for 120 min, depending on the experiment pre‐incubated with 5 μM CRID3, 30 or 50 μM VX765, or the vehicle DMSO, and subsequently treated with 20 μg/ml R837 for 120 min, unless otherwise specified. IL‐1β release into the tissue culture supernatant was determined by HTRF (b, h, k), cell death levels were determined measuring LDH release (c, i, l). Particle counts and size were determined using NTA. Relative particle counts were either normalized to the particle count upon LPS + R837 treatment (d) or normalized to the particle count upon LPS + DMSO + R837 treatment (g, j). For particle size distributions, particle counts were normalized to the total number of particles measured in each EV class (f). To visualize EVs, they were transferred to a carbon‐coated copper grid, stained with 2% aqueous uranyl acetate and subjected to transmission electron microscopy. Scale bar = 100 nm (e). (m, n), 10 · 10^6^ PMA‐differentiated doxycycline‐inducible gasdermin D KO THP‐1 macrophages per condition were primed with 200 ng/ml LPS for 120 min and subsequently stimulated with 20 μg/ml R837 for 120 min. Particle counts were determined using NTA. Relative particle counts were normalized to the particle count upon LPS + R837 treatment in no Dox cells (first black bar) in each EV class (m). IL‐1β release into the tissue culture supernatant was determined by HTRF (n). b, c, f−n, Pooled data from *n* = 3, each in technical triplicates, mean + SEM. d, Representative experiment from *n* = 2, each in technical triplicates, mean + SD. ns: not significant, *: *P‐*value < 0.05, **: *P‐*value < 0.01, ***: *P‐*value < 0.001, ****: *P‐*value < 0.0001.
Supplementary Fig. 2: Effects of CRID3, VX765, and GSDMD KO on EV release upon LPS + IFM, LPS + MSU, LPS + PrgI, LPS + R848, R848, and P3CSK4 stimulation. (a), Table summarizing cells, their stimulation and the read‐outs performed in experiments depicted in b−ag. MΦ: macrophage. (b—g), 10 · 10^6^ PMA‐differentiated WT THP‐1 macrophages were primed with 200 ng/ml LPS for 120 min and treated with the stimulus indicated for 90 min or 120 min (b−e) or treated with a stimulus for 24 h without prior priming (f, g). Particle sizes and counts were determined using NTA. For particle size distributions, particle counts were normalized to the total number of particles measured in each EV class. (h–s), 10 · 10^6^ PMA‐differentiated WT THP‐1 macrophages were primed with 200 ng/ml LPS for 120 min, pre‐incubated with 5 μM CRID3, 50 μM VX765, or the vehicle DMSO and subsequently treated with the stimulus indicated for 90 min or 120 min. Particle counts were determined using NTA. Relative particle counts were normalized to the particle count released upon LPS + DMSO + stimulus (black bar; h, k, n, q). IL‐1β release into the tissue culture supernatant was determined by HTRF (i, l, o, r), cell death levels were determined measuring LDH release (j, m, p, s). (t–y), 10 · 10^6^ PMA‐differentiated WT THP‐1 macrophages were pre‐incubated with either 5 μM CRID3, 50 μM VX765, or the vehicle DMSO. Subsequently, cells were stimulated with R848 or P3CSK4 for 24 h. Particle counts were determined using NTA. Relative particle counts were normalized to the particle count released upon DMSO + stimulus (black bar; t, w). IL‐1β release into the tissue culture supernatant was determined by HTRF (u, x), cell death levels were determined measuring LDH release (v, y). (z—ae), 10 · 10^6^ PMA‐differentiated doxycycline‐inducible gasdermin D KO THP‐1 macrophages were primed with 200 ng/ml LPS for 2 h and subsequently treated with the stimulus indicated for 90 min or 120 min. Particle counts were determined using NTA. Relative particle counts were normalized to the particle count upon LPS + stimulus treatment in no Dox cells (first black bar) in each EV class (z, ab, ad). IL‐1β release into the tissue culture supernatant was determined by HTRF (aa, ac, ae). (af, ag), 10 · 10^6^ PMA‐differentiated WT THP‐1 macrophages were primed with 200 ng/ml LPS for 120 min and left subsequently untreated for 120 min (af) or were left untreated for 24 h (ag). Particle sizes and counts were determined using NTA. For particle size distributions, particle counts were normalized to the total number of particles measured in each EV class. Pooled data from n = 3, each in technical triplicates, mean + SEM (b−y, ab−ag) or n = 2, each in technical triplicates, mean + SD (z, aa). ns: not significant, †: unadjusted *P*‐value < 0.05, *: *P‐*value < 0.05, **: *P‐*value < 0.01, ***: *P‐*value < 0.001, ****: *P‐*value < 0.0001.
Supplementary Fig. 3: Further characterization of the transcriptomics of EV content. (a), Number of replicate samples left for EV transcript analysis out of originally *n* = 5 per condition. (b), Multidimensional scaling (MDS) plot visualizing the relationship between the EV samples. (c), Gene set testing was performed using the camera function from the limma package. The top 40 cellular component GO terms in 10K versus SEC comparisons across all stimuli were plotted, ordered by average rank. (d), Number of transcripts significantly up‐ or down‐regulated comparing EVs released upon stimulation versus background (i.e., EVs released upon LPS priming or no treatment). (e, f), Venn diagrams depicting the overlap of significantly changed transcripts shared between LPS + nigericin, LPS + IFM, and LPS + R837 in 10K (e) and SEC EVs (f). (g, h), Venn diagrams depicting the overlap of significantly changed transcripts shared between LPS + MSU in 10K (g) and SEC EVs (h) and those transcripts commonly changed across inflammasome activators from (e) and (f) respectively.
Supplementary Fig. 4: Further characterization of the NLRP3 signature. (a), Number of transcripts commonly up‐ or down‐regulated in the inflammasome (Infl.) and NLRP3 signatures. Bars are coloured if transcripts were commonly changed across both signatures and both EV classes (black) or according to the NLRP3 signature. (b), Normalized log_2_ expression values of the *shokerbo* transcript across the samples indicated. (c), Normalized log_2_ expression values of the lncRNA *NONHSAT134003* across the samples indicated. (d), Gene set over‐representation analysis was performed using the egsea.ora function of the EGSEA package on significantly up‐regulated NLRP3 signature transcripts in 10K or SEC EVs. Plot shows all detected cellular component GO terms, with the top 30 GO terms for both EV subpopulations highlighted by colour. Grouping of highlighted GO terms was done in six categories: mitochondrion‐, ribosome‐, nucleus‐, ER‐, cytoskeleton‐associated GO terms, and all remaining ones (other). Dashed line indicates adjusted *P*‐value threshold of 0.05. (e, f), Transcripts significantly up‐ (e) or down‐regulated (f) within the NLRP3 signature were assessed regarding their transcript types using Ensembl BioMart and the locus type annotation provided by ThermoFisher. nc = non‐coding, lnc = long non‐coding. Adjusted *P‐*value threshold = 0.05. *: Adjusted *P‐*value < 0.05.
Supplementary Figure 5: NLRP3‐induced EVs are free of endotoxin but contain IL‐1β and induce similar effects in recipient cells independent of the EV‐inducing stimulus. (a), 4 · 10^6^ or 10 · 10^6^ PMA‐differentiated THP‐1 macrophages were stimulated as indicated and 2K, 10K, and SEC EVs were isolated from the tissue culture supernatant. Endotoxin quantification was performed using the Pierce Chromogenic Endotoxin Quant Kit. 1 EU/ml ≈ 0.1–0.2 ng/ml. Representative experiment of *n* = 2, technical duplicates, mean + SD. conc. = concentration. (b, c), During the EV transfer experiment (scheme depicted in Figure 5a), supernatants were taken after priming of recipient cells (b) and after EV transfer or full stimulation of recipient cells (c) to monitor cytokine release. TNFα and IL‐1β levels were quantified by HTRF. *n* = 5, each in technical triplicates, mean + SEM. (d), 6.5 · 10^6^, 4.5 · 10^6^ and 2 · 10^6^ PMA‐ differentiated THP‐1 macrophages were stimulated with LPS + nigericin to induce EV release. 2K, 10K and SEC EVs were isolated from the tissue culture supernatant. EVs were either lysed in 0.1 % Triton X‐100 (TX) or immediately subjected to IL‐1β HTRF. Pooled data of n = 3, each in technical triplicates, mean + SEM. (e), Comparison of 10K_LPS+Nig_ EV effect and 10K_LPS+R837_ EV effect on N3KO recipient cells. Significantly different transcripts are highlighted by color. Adjusted *P‐*value threshold = 0.05. ns: not significant, ****: *P‐*value < 0.0001.
Supplementary Fig. 6: 10K_LPS+Nig_ EV‐induced transcript promoters are enriched in predicted TF binding sites for IRFs, STATs, and NF‐кB. (a, b), List of the top enriched transcription factor binding sites (TFBSs) in promoters of genes that were significantly up‐regulated upon 10K_LPS+Nig_ transfer in N3KO as well as N3KO + N3 recipient cells (a) or specifically in N3KO + N3 recipient cells only (b). TFBS prediction was done using three different tools: CiiiDER, AME, and HOMER. TFBSs were ordered by their mean rank. Cluster specifies transcription factor cluster number according to JASPAR 2018. Not significant and missing (NA) TFBSs are displayed in grey. (c, d), t‐SNE plots of all TFs using the JASPAR motif distance table. TFs that were either enriched significantly upon 10K_LPS+Nig_ transfer in N3KO as well as N3KO + N3 recipient cells (c) or specifically in N3KO + N3 recipient cells only (d) are coloured by AME *p‐*value. Circles indicate NF‐κB TFs (cluster #26) and IRF TFs (cluster #31). (e), List of the top enriched transcription factor binding sites (TFBSs) in promoters of genes that were significantly up‐regulated upon LPS treatment in N3KO as well as N3KO + N3 recipient cells. TFBS prediction was done and displayed as described in a, b. (f), t‐SNE plots of all TFs using the JASPAR motif distance table. TFBSs are coloured by AME *P‐*values. Circles indicate NF‐κB TFs (cluster #26) and IRF TFs (cluster #31). *P‐*value threshold = 0.05.
Supplementary Fig. 7: Induction of ISGs by 10K EVs is independent of STING and is species‐specific. (a), Table summarizing cells, their stimulation and the read‐outs performed in experiments depicted in b−e. MΦ: macrophage. (b), Per condition, 3.25 · 10^5^ GM‐CSF‐differentiated human monocyte‐derived macrophages (hMDMs) were stimulated with 10K_LPS+Nig_ EVs released by WT THP‐1 macrophages or 5 · 10^3^ U/ml IFNβ for 15 h. RNA was isolated from recipient cells and subjected to qRT‐PCR. (c), EV release by PMA‐differentiated WT THP‐1 macrophages was induced by stimulation with LPS + nigericin (10K_LPS+Nig_ and SEC_LPS+Nig_). Per condition, 3.25 · 10^5^ PMA‐differentiated WT THP‐1 macrophages were pre‐incubated with 10 μM H‐151 (STING inhibitor) or vehicle control DMSO for 1 h. Subsequently, cells were stimulated with 10K_LPS+Nig_ or SEC_LPS+Nig_ EVs (40:1 EV donor cell to recipient cell ratio) or c‐di‐AMP for 15 h. RNA was isolated from recipient cells and subjected to qRT‐PCR. (d), Per condition, 3.25 · 10^5^ BMDMs of WT, STING KO, cGAS KO, and UNC93B KO mice were differentiated for 6 days using 20% L929 cell‐conditioned supernatant. Cells were left untreated or stimulated with 10K_LPS+Nig_ EVs released by WT THP‐1 macrophages or WT immortalized mouse macrophages (iMo; 40:1 EV donor cell to recipient cell ratio) for 15 h. RNA was isolated from recipient cells and subjected to qRT‐PCR. (e), Per condition, 3.25 · 10^5^ PMA‐differentiated WT macrophages (left) or WT immortalized mouse macrophages (right) were stimulated with EVs released by PMA‐differentiated WT macrophages or WT immortalized mouse macrophages upon LPS + nigericin (10K_LPS+Nig_) or LPS (10K_LPS_) treatment for 15 h. RNA was isolated from recipient cells and subjected to qRT‐PCR. d, Pooled data of *n* = 3 (b−d) or *n* = 4 (e), each in technical duplicates, mean ± SEM.
Supplementary Fig. 8: EV effect on bystander cells is dependent on the priming state of the cell. (a), Schematic diagram of the experiment. WT PMA‐differentiated THP‐1 macrophages were stimulated with LPS + nigericin or LPS + R837 to induce EV release. 8 · 10^4^ hMDMs per condition were primed with LPS or left untreated. Subsequently, recipient cells were co‐cultured with the EVs for 20 h at a 40:1 EV donor cell to EV recipient cell ratio or left untreated. (b), Tissue culture supernatants were taken after EV transfer and subjected to IL‐1β HTRF. (c), After EV transfer, cell death levels were determined by performing CTB assays on recipient cells. (d), Cytokine levels in EVs only were determined by TNFα and IL‐1β HTRF in the same volume cells were stimulated in *n* = 3, each in technical triplicates, mean + SEM. ns: not significant, *: *P‐*value < 0.05, ND = not detected.
