# Supplementary material for: Inflammasome‐induced extracellular vesicles harbour distinct RNA signatures and alter bystander macrophage responses
Source: J Extracell Vesicles. 2021 Aug 2;10(10):e12127. doi: 10.1002/jev2.12127 (PMC8329986; doi:10.1002/jev2.12127)
Supplement: Supplementary file 1 — Supplementary Fig. 1: EV release upon NLRP3 activation with LPS + R837 temporally correlates with IL‐1β release and is an NLRP3‐, caspase 1‐, and gasdermin D‐dependent event. (a), Table summarizing cells, their stimulation and the read‐outs performed in experiments depicted in b−n. MΦ: macrophage. (b–l), 10 · 106 PMA‐differentiated WT THP‐1 macrophages were primed with 200 ng/ml LPS for 120 min, depending on the experiment pre‐incubated with 5 μM CRID3, 30 or 50 μM VX765, or the vehicle DMSO, and subsequently treated with 20 μg/ml R837 for 120 min, unless otherwise specified. IL‐1β release into the tissue culture supernatant was determined by HTRF (b, h, k), cell death levels were determined measuring LDH release (c, i, l). Particle counts and size were determined using NTA. Relative particle counts were either normalized to the particle count upon LPS + R837 treatment (d) or normalized to the particle count upon LPS + DMSO + R837 treatment (g, j). For particle size distributions, particle counts were normalized to the total number of particles measured in each EV class (f). To visualize EVs, they were transferred to a carbon‐coated copper grid, stained with 2% aqueous uranyl acetate and subjected to transmission electron microscopy. Scale bar = 100 nm (e). (m, n), 10 · 106 PMA‐differentiated doxycycline‐inducible gasdermin D KO THP‐1 macrophages per condition were primed with 200 ng/ml LPS for 120 min and subsequently stimulated with 20 μg/ml R837 for 120 min. Particle counts were determined using NTA. Relative particle counts were normalized to the particle count upon LPS + R837 treatment in no Dox cells (first black bar) in each EV class (m). IL‐1β release into the tissue culture supernatant was determined by HTRF (n). b, c, f−n, Pooled data from n = 3, each in technical triplicates, mean + SEM. d, Representative experiment from n = 2, each in technical triplicates, mean + SD. ns: not significant, *: P‐value < 0.05, **: P‐value < 0.01, ***: P‐value < 0.001, ****: [file JEV2-10-e12127-s001.docx]

**
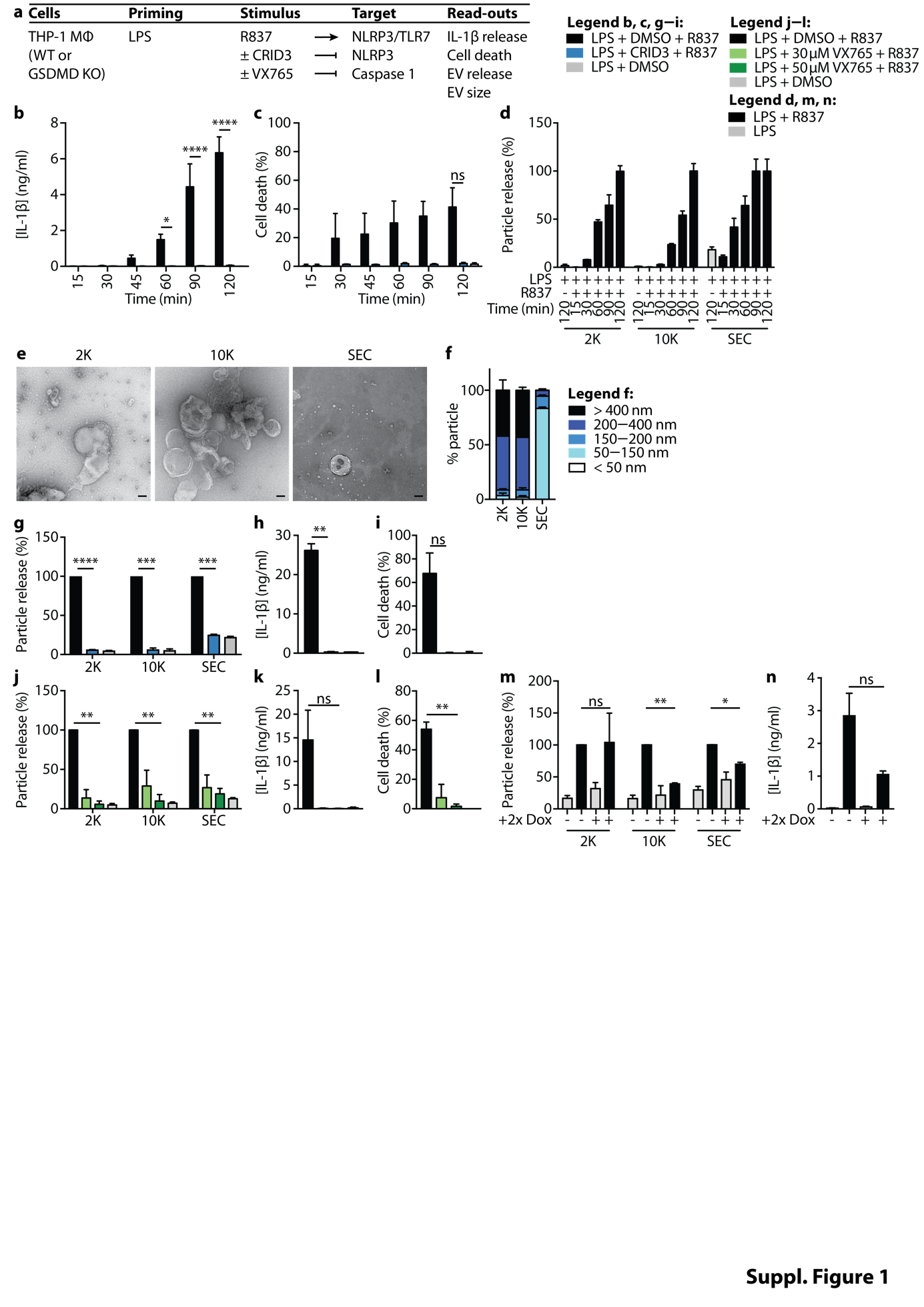

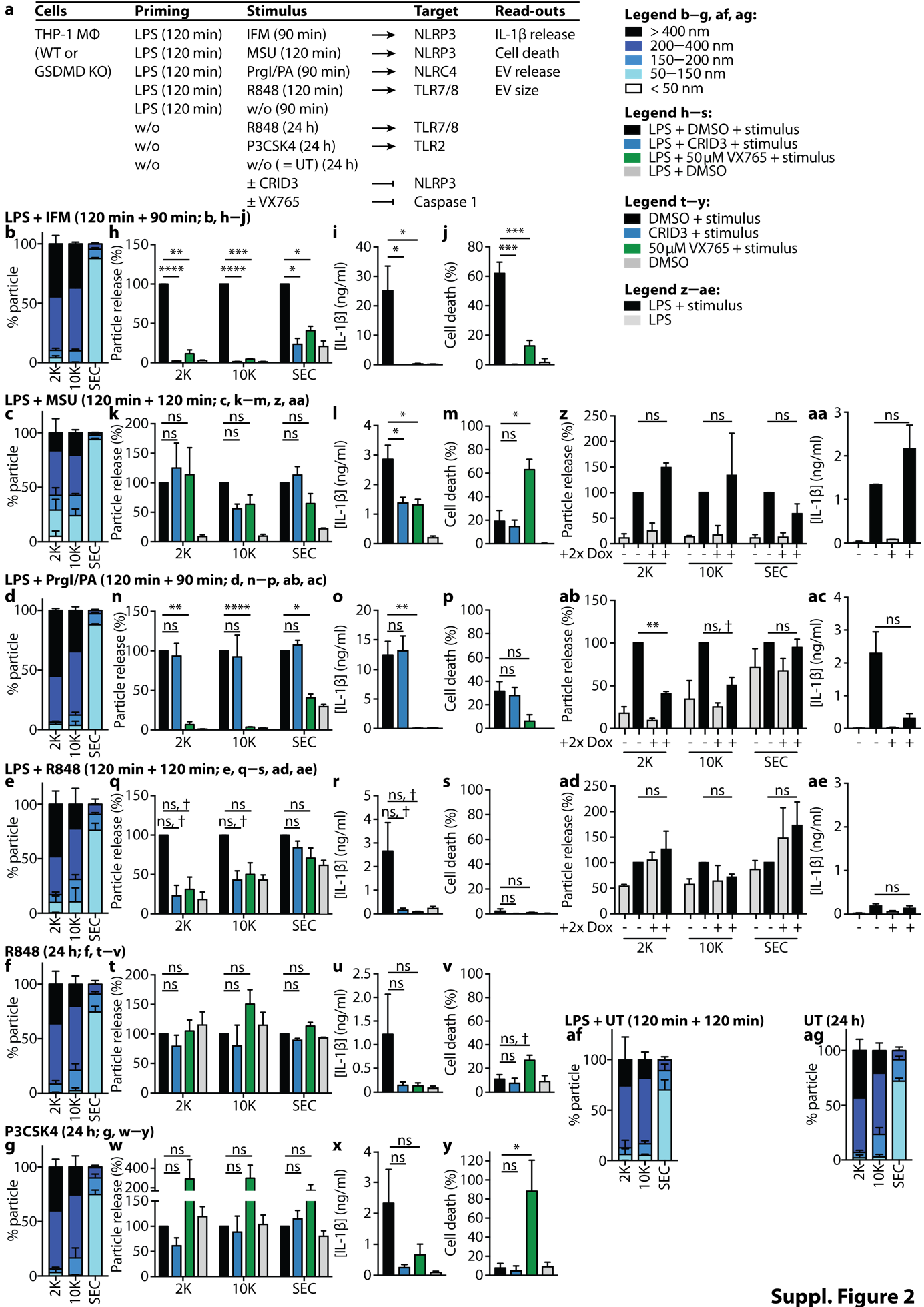
**


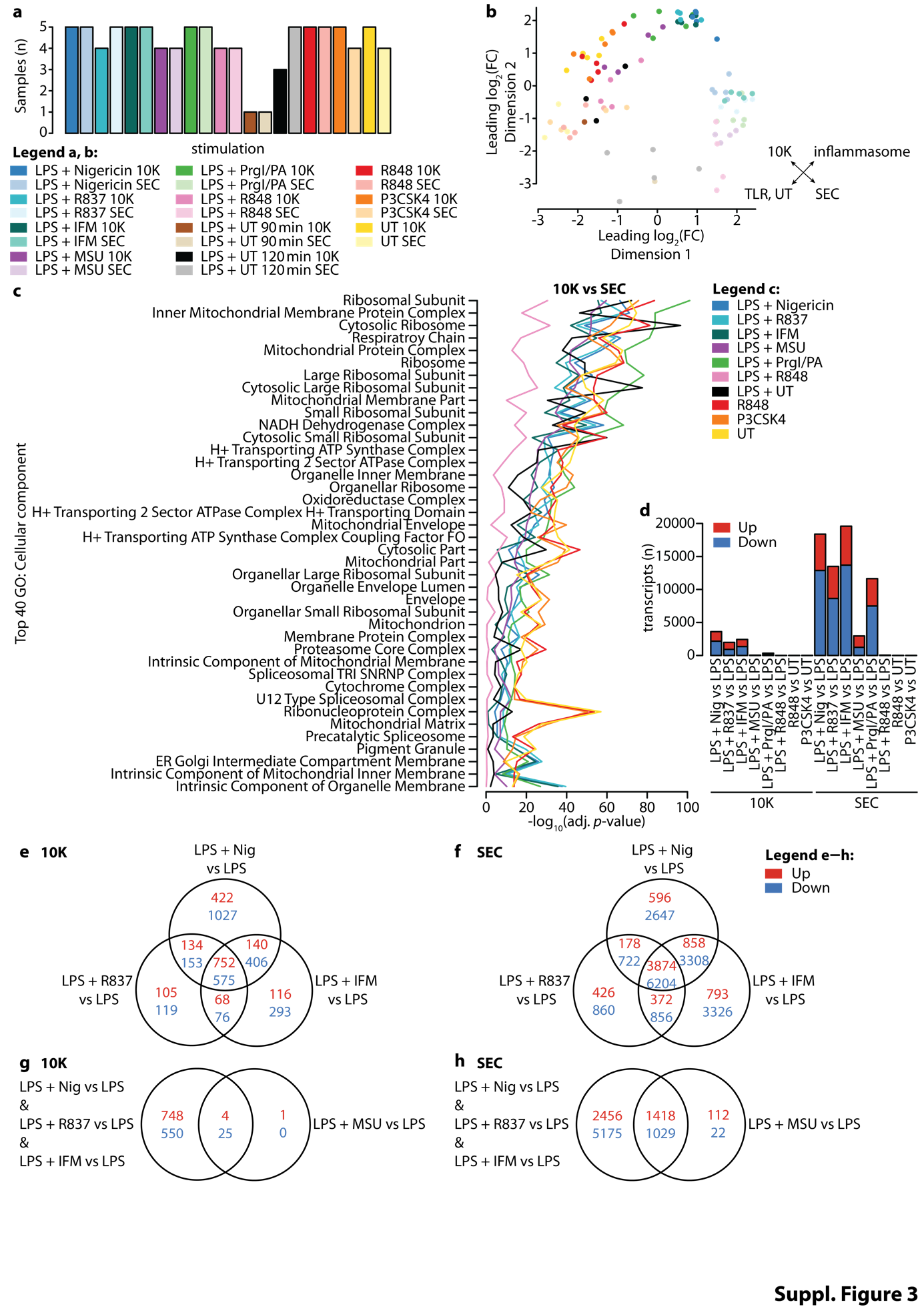


**
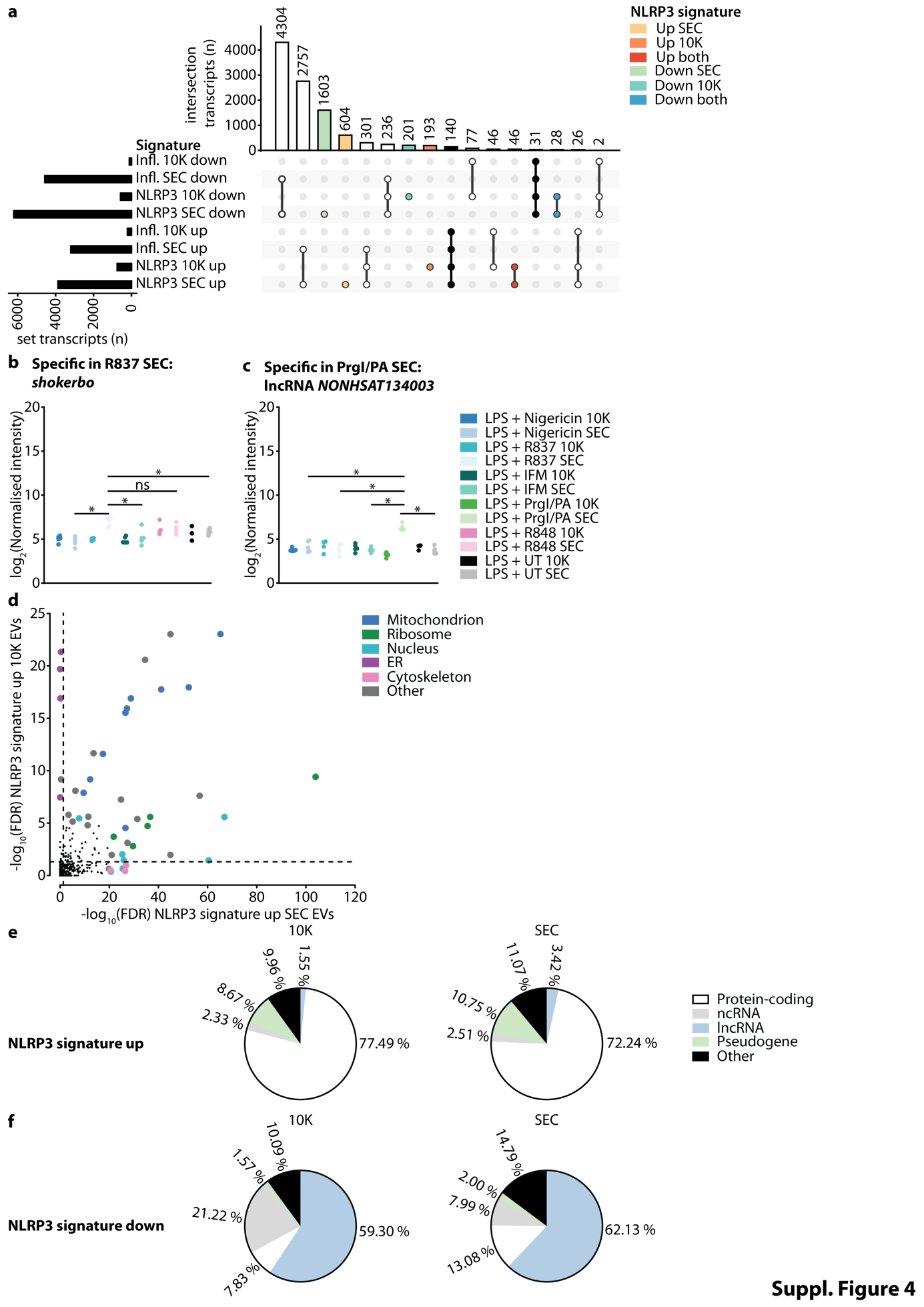
**

**
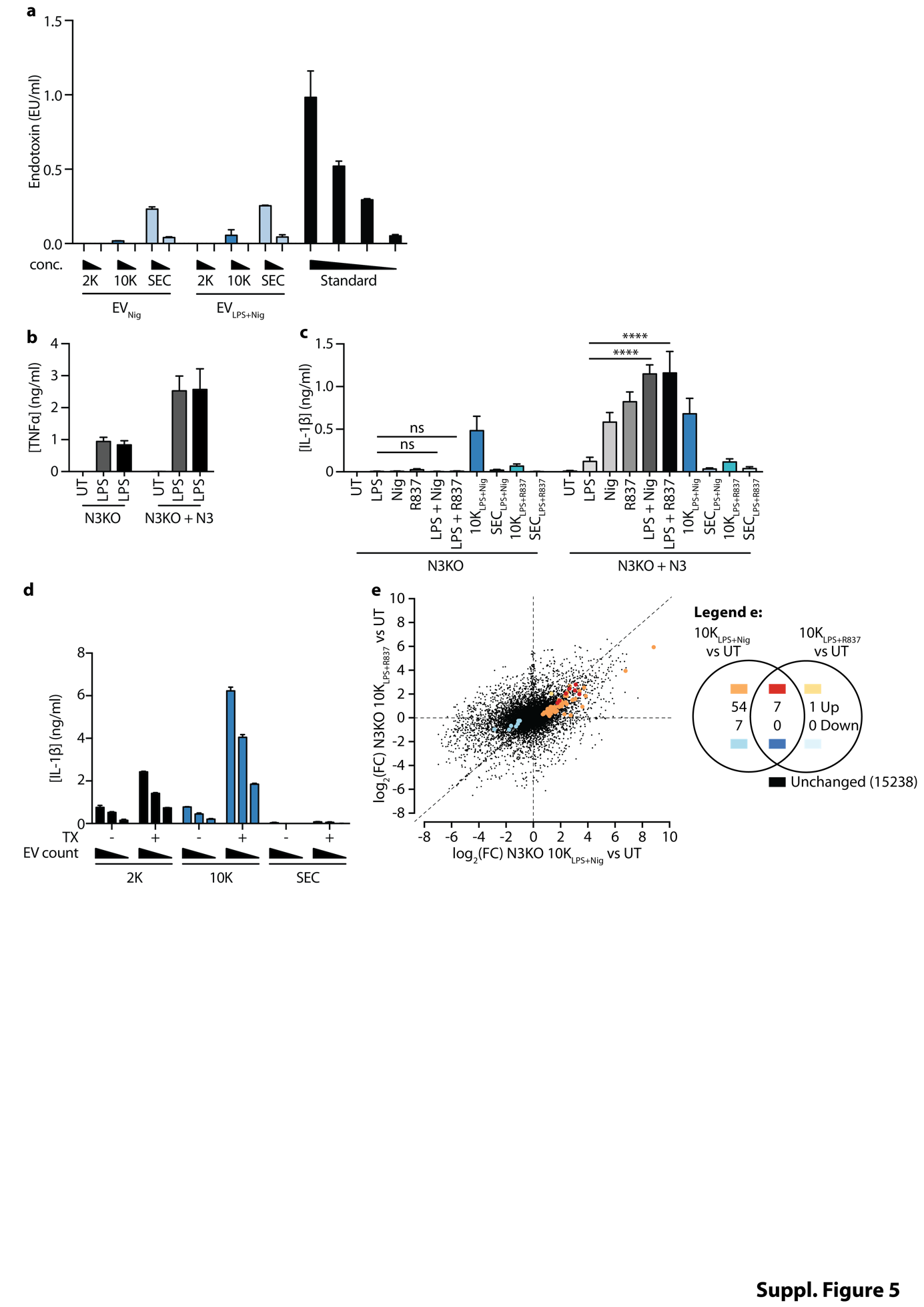
**

**
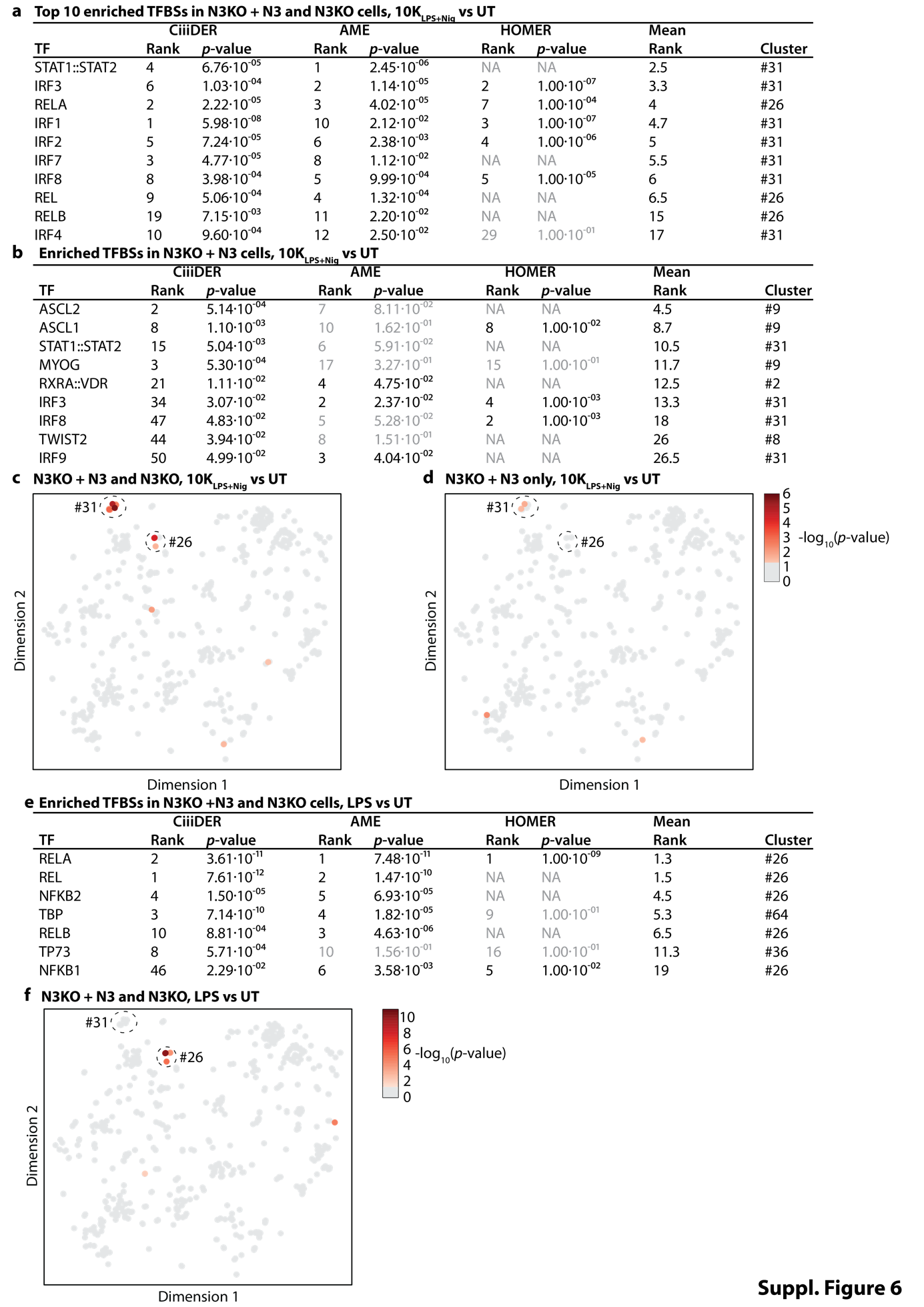
**

**
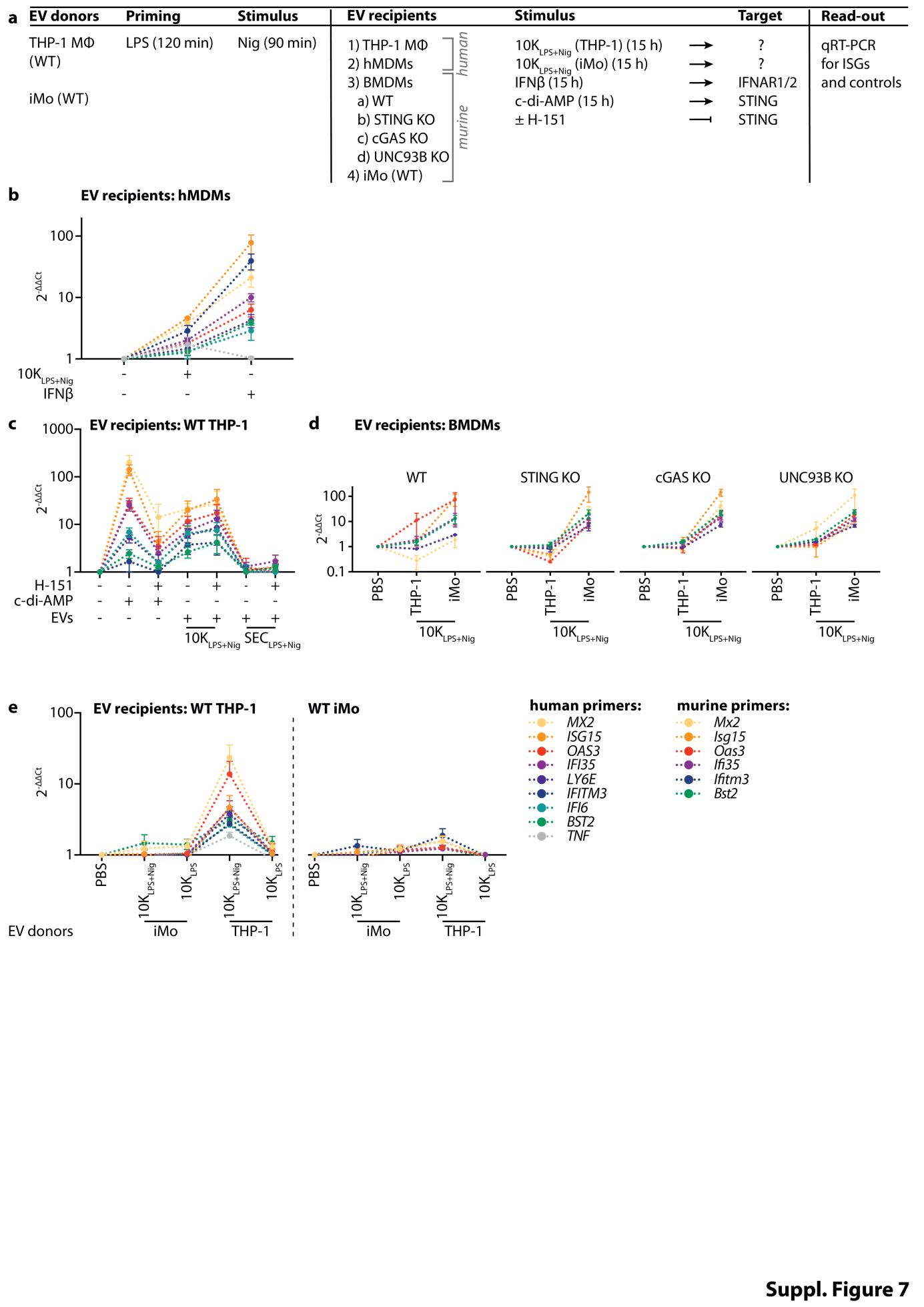
**

**
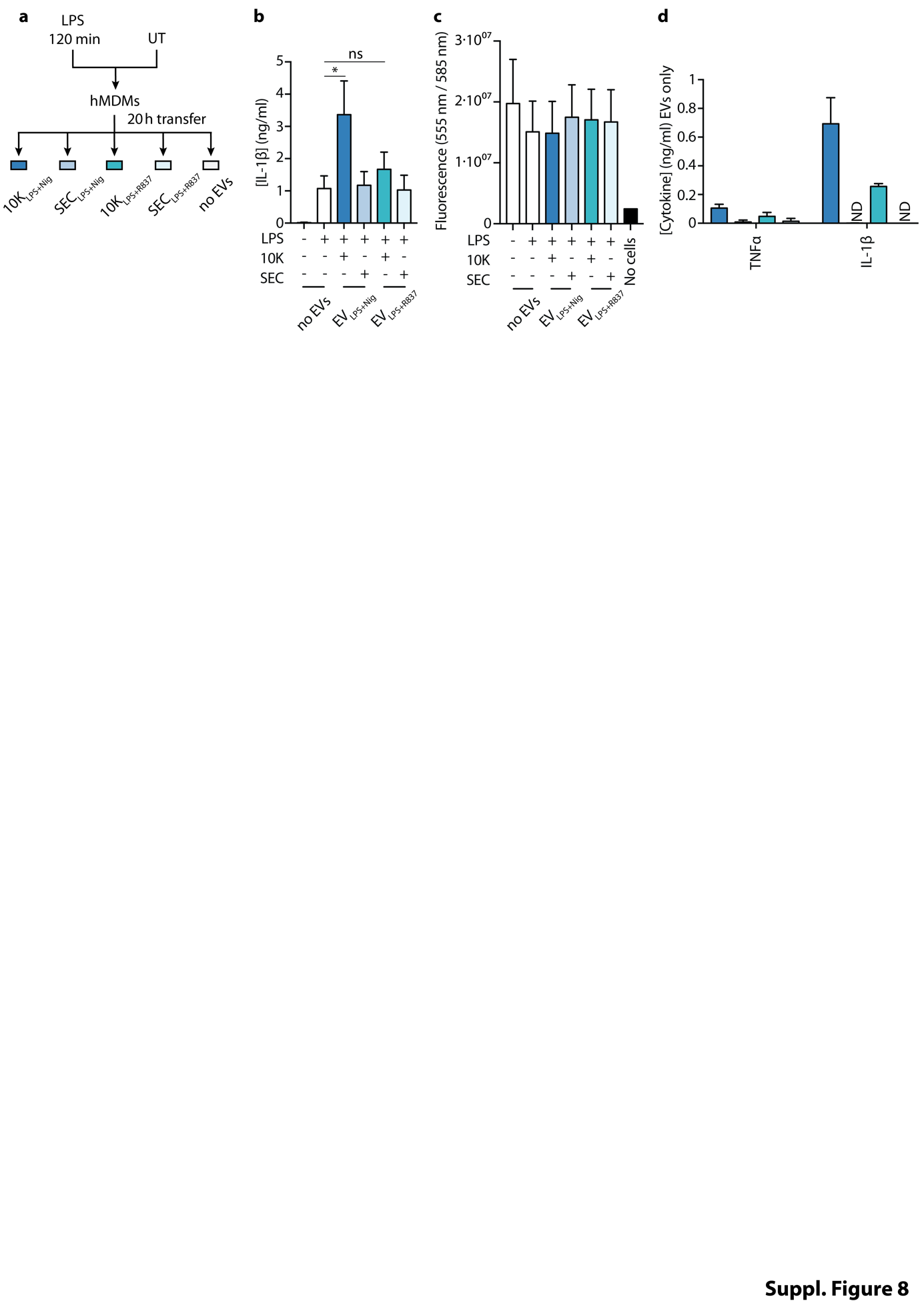
**

**Supplementary Fig. 1: EV release upon NLRP3 activation with LPS + R837 temporally correlates with IL-1β release and is an NLRP3-, caspase 1-, and gasdermin D-dependent event.**

**a,** Table summarizing cells, their stimulation and the read-outs performed in experiments depicted in b−n. MΦ: macrophage. **b−l,** 10 · 10^6^ PMA-differentiated WT THP-1 macrophages were primed with 200 ng/ml LPS for 120 min, depending on the experiment pre-incubated with 5 µM CRID3, 30 or 50 µM VX765, or the vehicle DMSO, and subsequently treated with 20 µg/ml R837 for 120 min, unless otherwise specified. IL-1β release into the tissue culture supernatant was determined by HTRF (b, h, k), cell death levels were determined measuring LDH release (c, i, l). Particle counts and size were determined using NTA. Relative particle counts were either normalized to the particle count upon LPS + R837 treatment (d) or normalized to the particle count upon LPS + DMSO + R837 treatment (g, j). For particle size distributions, particle counts were normalized to the total number of particles measured in each EV class (f). To visualize EVs, they were transferred to a carbon-coated copper grid, stained with 2% aqueous uranyl acetate and subjected to transmission electron microscopy. Scale bar = 100 nm (e). **m, n,** 10 · 10^6^ PMA-differentiated doxycycline-inducible gasdermin D KO THP-1 macrophages per condition were primed with 200 ng/ml LPS for 120 min and subsequently stimulated with 20 µg/ml R837 for 120 min. Particle counts were determined using NTA. Relative particle counts were normalized to the particle count upon LPS + R837 treatment in no Dox cells (first black bar) in each EV class (m). IL-1β release into the tissue culture supernatant was determined by HTRF (n). b, c, f−n, Pooled data from n = 3, each in technical triplicates, mean + SEM. d, Representative experiment from n = 2, each in technical triplicates, mean + SD. ns: not significant, *: *p-*value < 0.05, **: *p-*value < 0.01, ***: *p-*value < 0.001, ****: *p-*value < 0.0001.

**Supplementary Fig. 2: Effects of CRID3, VX765, and GSDMD KO on EV release upon LPS + IFM, LPS + MSU, LPS + PrgI, LPS + R848, R848, and P3CSK4 stimulation.**

**a,** Table summarizing cells, their stimulation and the read-outs performed in experiments depicted in b−ag. MΦ: macrophage. **b−g,** 10 · 10^6^ PMA-differentiated WT THP-1 macrophages were primed with 200 ng/ml LPS for 120 min and treated with the stimulus indicated for 90 min or 120 min (b−e) or treated with a stimulus for 24 h without prior priming (f, g). Particle sizes and counts were determined using NTA. For particle size distributions, particle counts were normalized to the total number of particles measured in each EV class. **h−s,** 10 · 10^6^ PMA-differentiated WT THP-1 macrophages were primed with 200 ng/ml LPS for 120 min, pre-incubated with 5 µM CRID3, 50 µM VX765, or the vehicle DMSO and subsequently treated with the stimulus indicated for 90 min or 120 min. Particle counts were determined using NTA. Relative particle counts were normalized to the particle count released upon LPS + DMSO + stimulus (black bar; h, k, n, q). IL-1β release into the tissue culture supernatant was determined by HTRF (i, l, o, r), cell death levels were determined measuring LDH release (j, m, p, s). **t−y,** 10 · 10^6^ PMA-differentiated WT THP-1 macrophages were pre-incubated with either 5 µM CRID3, 50 µM VX765, or the vehicle DMSO. Subsequently, cells were stimulated with R848 or P3CSK4 for 24 h. Particle counts were determined using NTA. Relative particle counts were normalized to the particle count released upon DMSO + stimulus (black bar; t, w). IL-1β release into the tissue culture supernatant was determined by HTRF (u, x), cell death levels were determined measuring LDH release (v, y). **z−ae,** 10 · 10^6^ PMA-differentiated doxycycline-inducible gasdermin D KO THP-1 macrophages were primed with 200 ng/ml LPS for 2 h and subsequently treated with the stimulus indicated for 90 min or 120 min. Particle counts were determined using NTA. Relative particle counts were normalized to the particle count upon LPS + stimulus treatment in no Dox cells (first black bar) in each EV class (z, ab, ad). IL-1β release into the tissue culture supernatant was determined by HTRF (aa, ac, ae). **af, ag,** 10 · 10^6^ PMA-differentiated WT THP-1 macrophages were primed with 200 ng/ml LPS for 120 min and left subsequently untreated for 120 min (af) or were left untreated for 24 h (ag). Particle sizes and counts were determined using NTA. For particle size distributions, particle counts were normalized to the total number of particles measured in each EV class. Pooled data from n = 3, each in technical triplicates, mean + SEM (b−y, ab−ag) or n = 2, each in technical triplicates, mean + SD (z, aa). ns: not significant, : unadjusted *p*-value < 0.05, *: *p-*value < 0.05, **: *p-*value < 0.01, ***: *p-*value < 0.001, ****: *p-*value < 0.0001.

**Supplementary Fig. 3: Further characterization of the transcriptomics of EV content.**

**a,** Number of replicate samples left for EV transcript analysis out of originally n = 5 per condition. **b,** Multidimensional scaling (MDS) plot visualizing the relationship between the EV samples. **c,** Gene set testing was performed using the camera function from the limma package. The top 40 cellular component GO terms in 10K vs SEC comparisons across all stimuli were plotted, ordered by average rank. **d,** Number of transcripts significantly up- or down-regulated comparing EVs released upon stimulation vs background (i.e., EVs released upon LPS priming or no treatment). **e, f,** Venn diagrams depicting the overlap of significantly changed transcripts shared between LPS + nigericin, LPS + IFM, and LPS + R837 in 10K (e) and SEC EVs (f). **g, h,** Venn diagrams depicting the overlap of significantly changed transcripts shared between LPS + MSU in 10K (g) and SEC EVs (h) and those transcripts commonly changed across inflammasome activators from (e) and (f) respectively.

**Supplementary Fig. 4: Further characterization of the NLRP3 signature.**

**a,** Number of transcripts commonly up- or down-regulated in the inflammasome (Infl.) and NLRP3 signatures. Bars are colored if transcripts were commonly changed across both signatures and both EV classes (black) or according to the NLRP3 signature. **b,** Normalized log_2_ expression values of the *shokerbo* transcript across the samples indicated. **c,** Normalized log_2_ expression values of the lncRNA *NONHSAT134003* across the samples indicated. **d,** Gene set over-representation analysis was performed using the egsea.ora function of the EGSEA package on significantly up-regulated NLRP3 signature transcripts in 10K or SEC EVs. Plot shows all detected cellular component GO terms, with the top 30 GO terms for both EV subpopulations highlighted by color. Grouping of highlighted GO terms was done in six categories: mitochondrion-, ribosome-, nucleus-, ER-, cytoskeleton-associated GO terms, and all remaining ones (other). Dashed line indicates adjusted *p-*value threshold of 0.05. **e, f,** Transcripts significantly up- (e) or down-regulated (f) within the NLRP3 signature were assessed regarding their transcript types using Ensembl BioMart and the locus type annotation provided by ThermoFisher. nc = non-coding, lnc = long non-coding. Adjusted *p-*value threshold = 0.05. *: Adjusted *p-*value < 0.05.

**Supplementary Fig. 5: NLRP3-induced EVs are free of endotoxin but contain IL-1β and induce similar effects in recipient cells independent of the EV-inducing stimulus.
a,** 4 · 10^6^ or 10 · 10^6^ PMA-differentiated THP-1 macrophages were stimulated as indicated and 2K, 10K, and SEC EVs were isolated from the tissue culture supernatant. Endotoxin quantification was performed using the Pierce Chromogenic Endotoxin Quant Kit. 1 EU/ml ≈ 0.1−0.2 ng/ml. Representative experiment of n = 2, technical duplicates, mean + SD. conc. = concentration. **b, c,** During the EV transfer experiment (scheme depicted in Fig. 5a), supernatants were taken after priming of recipient cells (b) and after EV transfer or full stimulation of recipient cells (c) to monitor cytokine release. TNFα and IL-1β levels were quantified by HTRF. n = 5, each in technical triplicates, mean + SEM. **d,** 6.5 · 10^6^, 4.5 · 10^6^ and 2 · 10^6^ PMA- differentiated THP-1 macrophages were stimulated with LPS + nigericin to induce EV release. 2K, 10K and SEC EVs were isolated from the tissue culture supernatant. EVs were either lysed in 0.1 % Triton X-100 (TX) or immediately subjected to IL-1β HTRF. Pooled data of n = 3, each in technical triplicates, mean + SEM. **e,** Comparison of 10K_LPS+Nig_ EV effect and 10K_LPS+R837_ EV effect on N3KO recipient cells. Significantly different transcripts are highlighted by color. Adjusted *p-*value threshold = 0.05. ns: not significant, ****: *p-*value < 0.0001.

**Supplementary Fig. 6: 10K_LPS+Nig_ EV-induced transcript promoters are enriched in predicted TF binding sites for IRFs, STATs, and NF-кB.**

**a, b,** List of the top enriched transcription factor binding sites (TFBSs) in promoters of genes that were significantly up-regulated upon 10K_LPS+Nig_ transfer in N3KO as well as N3KO + N3 recipient cells (a) or specifically in N3KO + N3 recipient cells only (b). TFBS prediction was done using three different tools: CiiiDER, AME, and HOMER. TFBSs were ordered by their mean rank. Cluster specifies transcription factor cluster number according to JASPAR 2018. Not significant and missing (NA) TFBSs are displayed in grey. **c, d,** t-SNE plots of all TFs using the JASPAR motif distance table. TFs that were either enriched significantly upon 10K_LPS+Nig_ transfer in N3KO as well as N3KO + N3 recipient cells (c) or specifically in N3KO + N3 recipient cells only (d) are colored by AME *p-*value. Circles indicate NF-κB TFs (cluster #26) and IRF TFs (cluster #31). **e,** List of the top enriched transcription factor binding sites (TFBSs) in promoters of genes that were significantly up-regulated upon LPS treatment in N3KO as well as N3KO + N3 recipient cells. TFBS prediction was done and displayed as described in a, b. **f,** t-SNE plots of all TFs using the JASPAR motif distance table. TFBSs are colored by AME *p-*values. Circles indicate NF-κB TFs (cluster #26) and IRF TFs (cluster #31). *p-*value threshold = 0.05.

**Supplementary Fig. 7: Induction of ISGs by 10K EVs is independent of STING and is species-specific.**

**a,** Table summarizing cells, their stimulation and the read-outs performed in experiments depicted in b−e. MΦ: macrophage. **b,** Per condition, 3.25 · 10^5^ GM-CSF-differentiated human monocyte-derived macrophages (hMDMs) were stimulated with 10K_LPS+Nig_ EVs released by WT THP-1 macrophages or 5 · 10^3^ U/ml IFNβ for 15 h. RNA was isolated from recipient cells and subjected to qRT-PCR. **c,** EV release by PMA-differentiated WT THP-1 macrophages was induced by stimulation with LPS + nigericin (10K_LPS+Nig_ and SEC_LPS+Nig_). Per condition, 3.25 · 10^5^ PMA-differentiated WT THP-1 macrophages were pre-incubated with 10 µM H-151 (STING inhibitor) or vehicle control DMSO for 1 h. Subsequently, cells were stimulated with 10K_LPS+Nig_ or SEC_LPS+Nig_ EVs (40:1 EV donor cell to recipient cell ratio) or c-di-AMP for 15 h. RNA was isolated from recipient cells and subjected to qRT-PCR. **d,** Per condition, 3.25 · 10^5^ BMDMs of WT, STING KO, cGAS KO, and UNC93B KO mice were differentiated for six days using 20% L929 cell-conditioned supernatant. Cells were left untreated or stimulated with 10K_LPS+Nig_ EVs released by WT THP-1 macrophages or WT immortalized mouse macrophages (iMo; 40:1 EV donor cell to recipient cell ratio) for 15 h. RNA was isolated from recipient cells and subjected to qRT-PCR. **e**, Per condition, 3.25 · 10^5^ PMA-differentiated WT macrophages (left) or WT immortalized mouse macrophages (right) were stimulated with EVs released by PMA-differentiated WT macrophages or WT immortalized mouse macrophages upon LPS + nigericin (10K_LPS+Nig_) or LPS (10K_LPS_) treatment for 15 h. RNA was isolated from recipient cells and subjected to qRT-PCR. d, Pooled data of n = 3 (b−d) or n = 4 (e), each in technical duplicates, mean ± SEM.

**Supplementary Fig. 8: EV effect on bystander cells is dependent on the priming state of the cell.**

**a,** Schematic diagram of the experiment. WT PMA-differentiated THP-1 macrophages were stimulated with LPS + nigericin or LPS + R837 to induce EV release. 8 · 10^4^ hMDMs per condition were primed with LPS or left untreated. Subsequently, recipient cells were co-cultured with the EVs for 20 h at a 40:1 EV donor cell to EV recipient cell ratio or left untreated. **b,** Tissue culture supernatants were taken after EV transfer and subjected to IL-1β HTRF. **c,** After EV transfer, cell death levels were determined by performing CTB assays on recipient cells. **d,** Cytokine levels in EVs only were determined by TNFα and IL-1β HTRF in the same volume cells were stimulated in. n = 3, each in technical triplicates, mean + SEM.

ns: not significant, *: *p-*value < 0.05, ND = not detected.
